# Assembly of Dynamic Gated and Cascaded Transient DNAzyme Networks

**DOI:** 10.1021/acsnano.1c11631

**Published:** 2022-03-16

**Authors:** Jiantong Dong, Yu Ouyang, Jianbang Wang, Michael P. O’Hagan, Itamar Willner

**Affiliations:** Institute of Chemistry, Center for Nanoscience and Nanotechnology, The Hebrew University of Jerusalem, Jerusalem 91904, Israel

**Keywords:** G-quadruplex, nicking enzyme, out-of-equilibrium, dissipative, DNA network, machinery, DNA nanotechnology

## Abstract

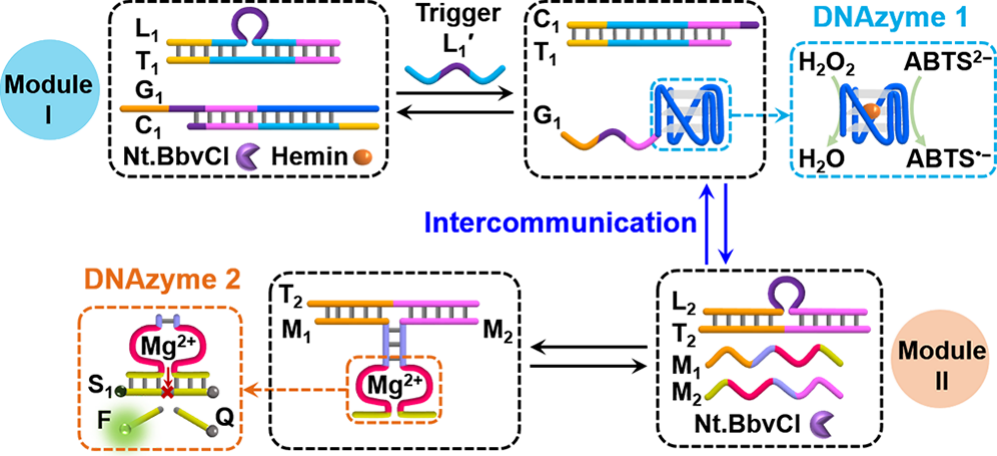

The dynamic transient formation and depletion of G-quadruplexes regulate gene replication and transcription. This process was found to be related to various diseases such as cancer and premature aging. We report on the engineering of nucleic acid modules revealing dynamic, transient assembly and disassembly of G-quadruplex structures and G-quadruplex-based DNAzymes, gated transient processes, and cascaded dynamic transient reactions that involve G-quadruplex and DNAzyme structures. The dynamic transient processes are driven by functional DNA reaction modules activated by a fuel strand and guided toward dissipative operation by a nicking enzyme (Nt.BbvCI). The dynamic networks were further characterized by computational simulation of the experiments using kinetic models, allowing us to predict the dynamic performance of the networks under different auxiliary conditions applied to the systems. The systems reported herein could provide functional DNA machineries for the spatiotemporal control of G-quadruplex structures perturbing gene expression and thus provide a therapeutic means for related emergent diseases.

## Introduction

Different biological processes such as cell proliferation,^1,2^ cell motility,^3,4^ and signal promotion^5^ represent spatiotemporal reactions proceeding under dissipative, out-of-equilibrium conditions. Substantial recent research efforts are directed toward the development of synthetic systems emulating such processes. For example, the GTP-driven growth and division of protein fibrils in coacervated droplets was suggested as a model system mimicking spatiotemporal cell division.^6^ Also, the dissipative carbodiimide-fueled synthesis of anhydrides^7^ and the transient assembly and disassembly of fibers by the catalyzed hydrolysis of peptides^8^ were demonstrated in systems operating out-of-equilibrium.

Within these efforts, the information encoded in nucleic acids provides versatile means to assemble spatiotemporal reaction networks and circuitries. The possibilities to control duplex strand displacement processes by fuel/antifuel strands dictated by the stability of the duplexes,^9,10^ the reconfiguration of triplex nucleic acids through strand displacement or auxiliary triggers,^11^ e.g., pH, the use of photoisomerizable intercalators, such as *trans*/*cis*-azobenzene, to assemble/disassemble nucleic acid duplexes,^12−14^ and the many available enzymes to cleave or ligate nucleic acids, such as endonucleases,^15,16^ nickases,^17^ exonucleases,^18,19^ DNAase,^20^ ligase,^21−23^ or biocatalytic nucleic acids (DNAzymes),^24−28^ provide a rich arsenal of functional reconfiguration motives. Besides using these molecular tools to design DNA-based switches^29^ and machines,^30−35^ dynamic networks and circuitries, such as a synthetic transcriptional clock,^36^ transcriptional oscillators,^37^ bistable transcriptional switches,^38^ and transcriptional regulatory networks^39^ were demonstrated. Also, wired small DNA templates were cascaded to yield dynamically controlled oscillatory outputs,^40^ and the dynamic out-of-equilibrium operations of such systems have been suggested to mimic natural ecosystems.^41^ In particular, DNA reaction modules operating transient, out-of-equilibrium, processes were recently developed. For example, the formation of a ligand-aptamer complex, e.g., the AMP-aptamer complex, and its subsequent biocatalytic separation resulted in the assembly and dynamic depletion of an aptamer-ligand complex as a reaction intermediate.^42^ In addition, different enzymes such as endonucleases,^43^ nicking enzymes,^44^ or synthetic catalytic nucleic acids, DNAzymes,^45^ were applied as catalysts to control the dynamic transient reconfiguration of DNA networks that stimulate the assembly and dissipative depletion of DNA structures. Biocatalytically driven gated transient systems and dissipative cascades^44^ were demonstrated, and the ATP-fueled transient ligation of DNAzyme subunits to yield catalytic DNAzymes was reported.^46^ In addition, the biocatalytically driven transient reconfiguration of constitutional dynamic networks was reported,^47^ and a dynamic transient feedback driven DNA network effecting the synthesis of oligonucleotides was coupled to giant membrane vesicles, thus acting as a protocell.^48^ The triggered formation and dissipation of the biopolymer intermediate demonstrated signal-responsive adaptive properties of the protocell, mimicking cellular homeostasis. Different applications of transient DNA networks were suggested, such as the temporary uptake and release of loads^49,50^ and the transient control over the optical properties of nucleic acid functionalized Au nanoparticles, semiconductor quantum dots or metal nanoclusters through their transient aggregation and disaggregation.^51^

G-quadruplexes attract substantial research interest as functional reconfigurable nucleic acid based structures. DNA switches^52−55^ and machines^56−60^ relying on the reconfiguration of G-quadruplexes and the switchable catalytic activities of hemin/G-quadruplex DNAzymes,^61^ have been a subject of extensive research. Different applications of reconfigurable G-quadruplex nanostructures were addressed including their use as reversible gating units of drug-loaded carriers,^62,63^ functional units controlling the stiffness of hydrogel matrices for shape-memory and controlled drug release,^64,65^ and the self-organization of photodynamic therapeutic agents.^66,67^ The dynamic transient formation and depletion of G-quadruplexes regulate the replication and transcription of genes,^68−73^ and the dynamic folding of G-quadruplexes was found to be important in the telomerase-stimulated synthesis of telomeres.^74^ Indeed, perturbing the folding and unfolding dynamics of G-quadruplexes was found to be related to various human diseases caused by genomic instability, such as premature aging and cancer.^75^ Realizing that G-quadruplexes are unfolded in nature by helicase and that helicase efficiency could affect the spatiotemporal formation and disassembly of G-quadruplexes leading to these respective biological disorders, the development of synthetic dynamic routes to form and unwind G-quadruplexes could provide a therapeutic means for G-quadruplex-related diseases.

In the present study, we introduce nucleic acid systems demonstrating the dynamic transient assembly/disassembly and operation of G-quadruplex structures. We discuss means to control the dynamic processes by auxiliary stimuli, such as the concentrations of the triggering stimuli and the accompanying biocatalysts and strategies to guide the dynamic transitions of the systems by controlling the effects of inhibitors on the gating of the process. In particular, we demonstrate the integration of the dynamic reconfiguration of the G-quadruplex within a cascaded transformation involving information transfer and dynamic intercommunication between two dissipative transient systems.

## Results and Discussion

**Figure 1:**
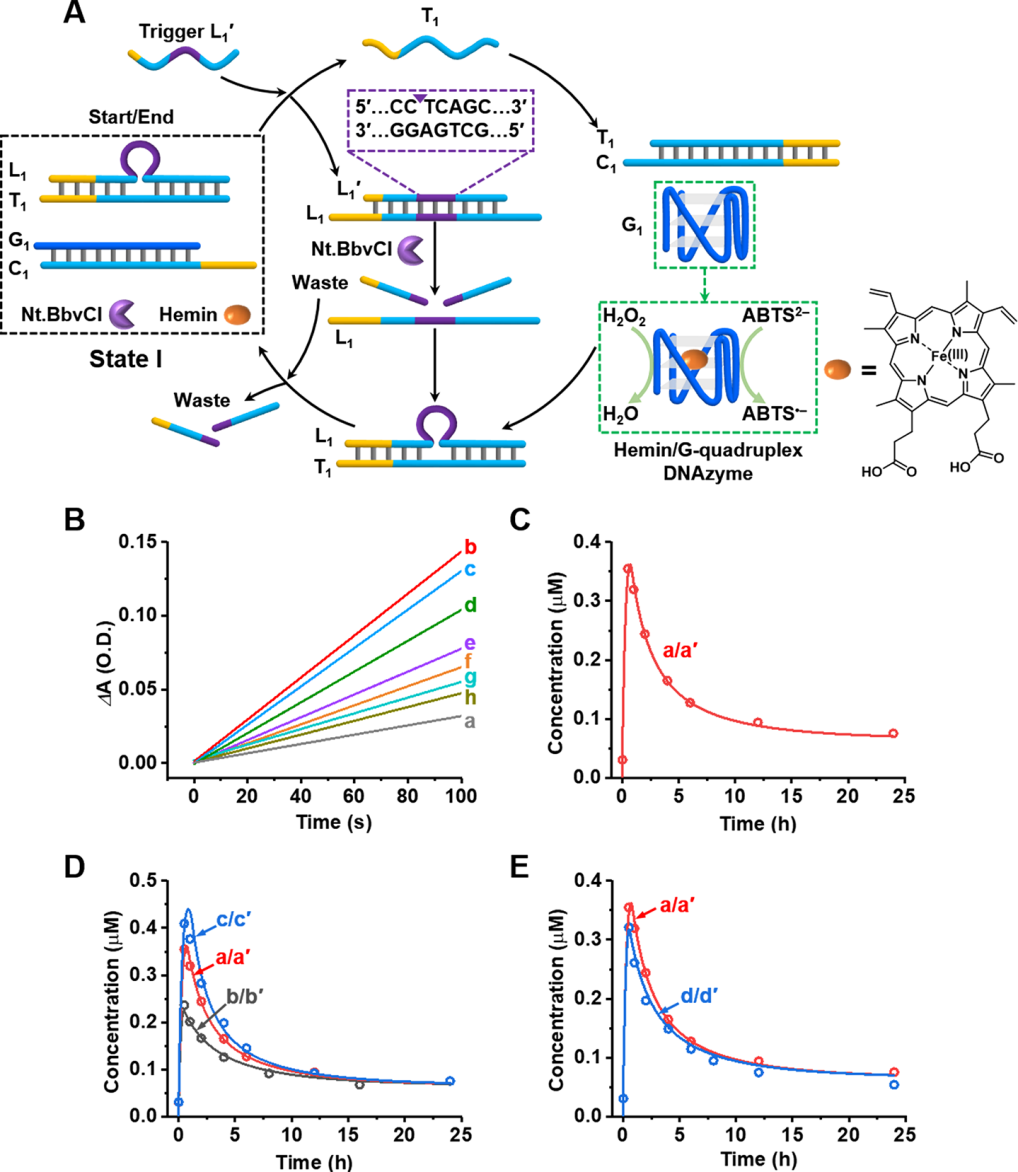
(A) Schematic illustration of the reaction module for the transient formation and depletion of the hemin/G-quadruplex DNAzyme. (B) Time-dependent absorbance changes of ABTS^•–^ generated by the hemin/G-quadruplex DNAzyme formed at different time-intervals of the transient operation of the reaction module: (a) 0 h, (b) 0.5 h, (c) 1 h, (d) 2 h, (e) 4 h, (f) 6 h, (g) 12 h, and (h) 24 h. (C) Dots, curve a; transient concentrations of the hemin/G-quadruplex DNAzyme; solid line, curve a′, computationally fitted transient concentrations of the DNAzyme using the kinetic model of the reaction module (Figure S2). Experimental and simulated results correspond to the reaction module consisting of [L_1_/T_1_] = 1 μM, [G_1_/C_1_] = 1 μM, [hemin] = 1 μM, [Nt.BbvCI] = 0.046 μM, and [L_1_′] = 4 μM. (D) Characterization of the transient behavior of the reaction module state I, under different conditions: curves a/a′, experimental and computational results presented in (C); curves b/b′, computationally predicted results (solid line, b′) and experimentally validated results (dots, b) in the presence of [L_1_′] = 2 μM; curves c/c′, computationally predicted results (solid line, c′) and experimentally validated results (dots, c) in the presence of [L_1_′] = 6 μM. All other conditions are the same as those described in (C). (E) Curves a/a′ are the same as those described in (C); solid line, curve d′, computationally predicted results in the presence of [Nt.BbvCI] = 0.069 μM; dots, curve d, experimentally validated results. All other conditions are the same as those described in (C).

Figure 1A depicts the transient assembly of a hemin/G-quadruplex DNAzyme structure. The reaction module, state I, includes two duplexes, L_1_/T_1_ and G_1_/C_1_, and nicking enzyme Nt.BbvCI, which acts as catalyst that controls the transient dissipative process. Hemin is added as an auxiliary effector to the reaction module. The strand G_1_ in the duplex G_1_/C_1_ consists of a guanosine-rich sequence that under appropriate conditions can assemble into a G-quadruplex. Subjecting the reaction module to trigger L_1_′ displaces duplex L_1_/T_1_ to yield duplex L_1_/L_1_′. The released strand T_1_ displaces duplex G_1_/C_1_ to yield duplex T_1_/C_1_ while releasing the strand G_1_ that assembles in the presence of K^+^ ions and hemin into the hemin/G-quadruplex DNAzyme. Duplex L_1_/L_1_′ is engineered to be cleaved by nicking enzyme Nt.BbvCI resulting in the cleavage of L_1_′ and the separation of L_1_. The released strand L_1_ then displaces duplex T_1_/C_1_, releasing C_1_ which disassembles the G-quadruplex through duplex formation to regenerate the initial reaction module, state I. Thus, the dynamic events proceeding in the network lead to the transient assembly and dissipative depletion of the hemin/G-quadruplex DNAzyme. The hemin/G-quadruplex DNAzyme catalyzed oxidation of 2,2′-azino-bis(3-ethylbenzothiazoline-6-sulfonic acid) (ABTS^2–^) to the colored ABTS^•–^ (λ = 420 nm) in the presence of hydrogen peroxide (H_2_O_2_), provides a readout signal for the transient formation and depletion of the DNAzyme. Figure 1B depicts the time-dependent absorbance changes of ABTS^•–^ formation by the hemin/G-quadruplex DNAzyme at different time intervals of the transient dynamic operation of the dissipative machinery shown in Figure 1A. Using an appropriate calibration curve, relating the time-dependent absorbance changes of ABTS^•–^ to known standard concentrations of the hemin/G-quadruplex DNAzyme, Figure S1, the transient concentration changes of the hemin/G-quadruplex DNAzyme were derived (Figure 1C, dots). A kinetics model that accounts for the different steps involved in the transient process was formulated (Figure S2) and solved computationally to determine the rate constants that describe the experimental results. The best-fit curve overlaying the experimental result is shown in Figure 1C (curve a′). The derived computational rate constants are summarized in Table S1. For a detailed description of the procedure for the computational simulations presented in the study, see the Supporting Information, page S6. The computationally simulated results are valuable to predict behavior of the system under different auxiliary experimental conditions. Realizing that the dynamic experiments shown in Figure 1C were obtained in the presence of L_1_′ (4 μM) and Nt.BbvCI (0.046 μM), the derived transient behavior of the system at two concentrations of L_1_′ (2 and 6 μM) and a constant concentration of Nt.BbvCI (0.046 μM) were displayed in curves b′ and c′ (Figure 1 D). The predicted dynamic behavior was then experimentally validated, as shown with dots in curves b and c. Similarly, Figure 1E depicts curve d′ that predicts the transient behavior of the system in the presence of a higher concentration of the nicking enzyme (0.069 μM) and L_1_′ (4 μM), and the dots (curve d) represent the experimental validation of the predicted curve. (For the experimental raw data leading to the results displayed in Figure 1D,E, see Figure S3.) Very good agreement between the predicted transient dynamic kinetics patterns of the systems at different auxiliary conditions and the experimental results is demonstrated, indicating the success of the computational modeling of the kinetics of the complex dynamic machinery. The formation and depletion of the G-quadruplex in the system was further supported by circular dichroism (CD) experiments (see Figure S4 and the accompanying discussion). In addition, the transient operation of the system shown in Figure 1 was further supported by quantitative gel electrophoretic experiments following the transient depletion and recovery of constituents L_1_/T_1_ and G_1_/C_1_ (see Figure S5 and the accompanying discussion).

**Figure 2:**
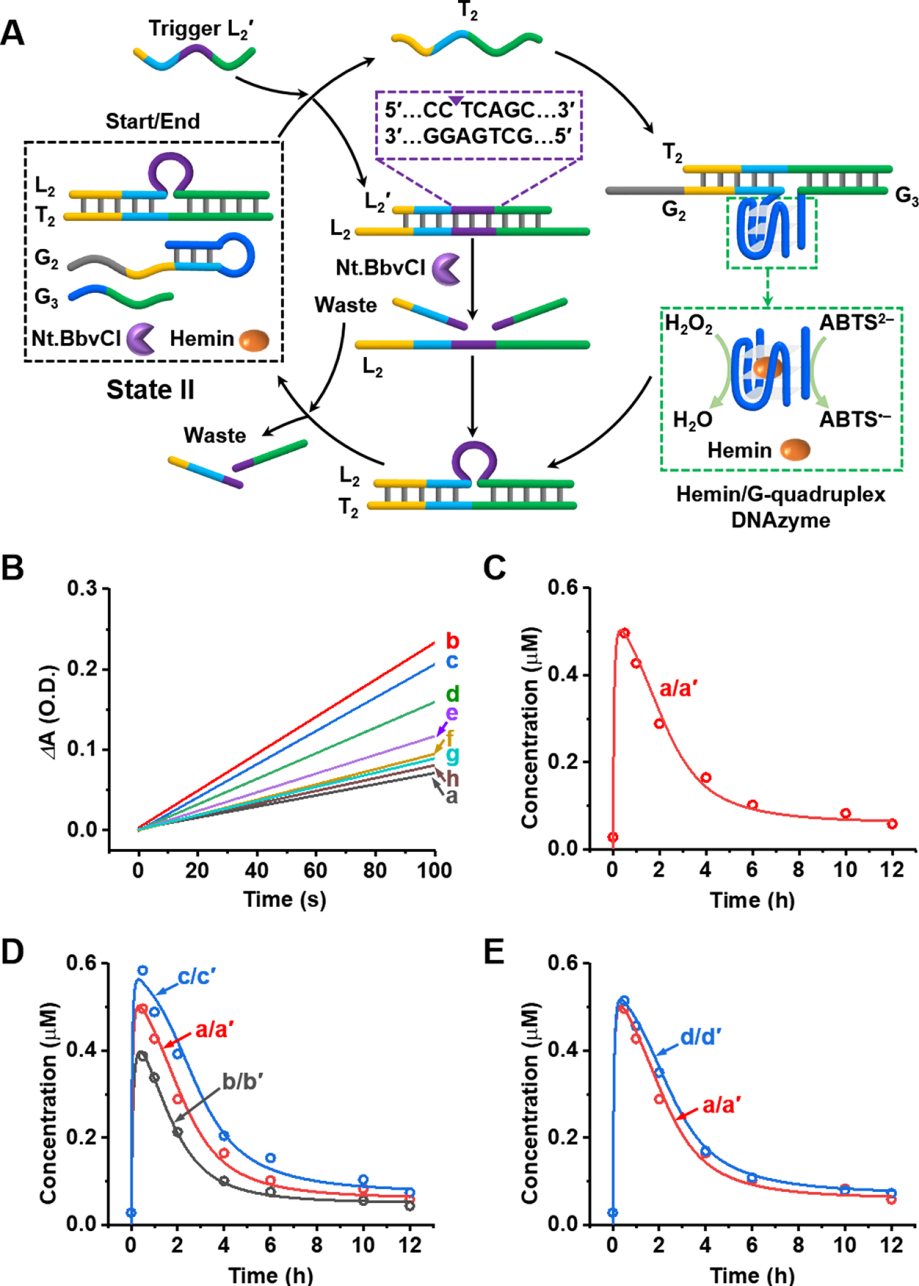
(A) Schematic diagram of the reaction module leading to the transient formation and depletion of the hemin/G-quadruplex DNAzyme composed of two subunit strands. (B) Time-dependent absorbance changes of ABTS^•–^ generated by the hemin/G-quadruplex T_2_/G_2_+G_3_ DNAzyme formed at different time intervals of the transient operation of the reaction module: (a) 0 h, (b) 0.5 h, (c) 1 h, (d) 2 h, (e) 4 h, (f) 6 h, (g) 10 h, (h) 12 h. (C) Dots, curve a, transient concentrations of the hemin/G-quadruplex T_2_/G_2_+G_3_ DNAzyme; solid line, curve a′, computationally fitted transient concentrations of the DNAzyme upon using the kinetic model of the reaction module (Figure S8). Experimental and fitted results correspond to the reaction module consisting of [L_2_/T_2_] = 1 μM, [G_2_] = 1 μM, [G_3_] = 1 μM, [hemin] = 1 μM, [Nt.BbvCI] = 0.069 μM, and [L_1_′] = 4 μM. (D) Characterization of the transient behavior of the reaction module under different conditions: curves a/a′, experimental and computational results presented in (C); curves b/b′, computationally predicted results (solid line, b′) and experimentally validated results (dots, b) in the presence of [L_2_′] = 2 μM; curves c/c′, computationally predicted results (solid line, c′) and experimentally validated results (dots, c) in the presence of [L_2_′] = 6 μM. All other conditions are the same as those in (C). (E) Curves a/a′ are the same as those described in (C); solid curve d′, computationally predicted results in the presence of [Nt.BbvCI] = 0.046 μM; dots, curve d, experimentally validated results. All other conditions are the same as those described in (C).

A second reaction module demonstrating the transient dynamic assembly of a supramolecular hemin/G-quadruplex, consisting of two G subunits, is depicted in Figure 2A. The reaction module in state II is composed of duplex L_2_/T_2_, hairpin structure G_2_, and single-strand G_3_ as constituents, nicking enzyme Nt.BbvCI as the participating catalyst, and hemin as the DNAzyme cofactor. Strands G_2_ and G_3_ include guanosine-rich sequences (blue) that act as subunits that assemble, under appropriate conditions, into the hemin/G-quadruplex supramolecular DNAzyme. Subjecting the reaction module in state II to trigger strand L_2_′ displaces duplex L_2_/T_2_ to yield L_2_/L_2_′, and the released strand, T_2_, opens hairpin G_2_ while simultaneously bridging the strands G_2_/G_3_ and promoting their self-assembly into the hemin/G-quadruplex DNAzyme. Duplex L_2_/L_2_′ is, however, engineered to include the nicking site in strand L_2_′ to be cleaved by Nt.BbvCI. Cleavage of L_2_′ leads to the release of L_2_ that displaces strand T_2_ from supramolecular complex T_2_/G_2_+G_3_, leading to separation of the hemin/G-quadruplex DNAzyme and to the eventual recovery of the rest module in state II. Thus, the network displayed in Figure 2A leads to the dynamic, transient formation and depletion of the supramolecular hemin/G-quadruplex DNAzyme structure. The dynamic behavior of the system was probed by the DNAzyme-catalyzed oxidation of ABTS^2–^ to ABTS^•–^. Figure 2B depicts the time-dependent absorbance changes of ABTS^•–^ generated by the dynamically formed hemin/G-quadruplex DNAzyme samples after different time intervals following the activation of the transient system. Using an appropriate calibration curve, relating the time-dependent absorbance changes of ABTS^•–^ to known concentrations of the supramolecular hemin/G-quadruplex (Figure S6), the concentrations of the transiently formed and depleted DNAzyme complex T_2_/G_2_+G_3_ were evaluated, Figure 2C (dotted profile). The full absorption spectra of ABTS^•–^ generated after a fixed time interval of 100 s by the samples withdrawn at differed time intervals following the activation of the transient system are presented in Figure S7A. The full absorption spectra of ABTS^•–^ generated after 100 s by known concentrations of the DNAzyme T_2_/G_2_+G_3_ are presented in Figure S7B. The absorbance values at λ = 420 nm were used to derive a respective calibration curve (Figure S7C), following which the absorbance values recorded for the transient samples at λ = 420 nm, ε = 36 000 /(M·cm) (Figure S7A), enabled the derivation of the transient concentrations of T_2_/G_2_+G_3_ generated in the transient system (Figure S7D). The kinetic model corresponding to the transient scheme depicted in Figure 2A was formulated (Figure S8). The computationally simulated kinetic curve is presented as the solid curve (red) overlaid on the experimental data, Figure 2C. The derived rate constants (Table S2) were then used to predict the behavior of the network at different auxiliary conditions, and the predicted results were experimentally validated (curves b/b′, c/c′, and d/d′ in Figure 2D,E; the raw results are displayed in Figure S9). The formation and depletion of the supramolecular G-quadruplex in the system was further supported by CD experiments (see Figure S10 and the accompanying discussion). Additionally, the transient operation of the system was supported by quantitative gel electrophoretic experiments following the transient depletion and recovery of the constituents L_2_/T_2_, Figure S11 and the accompanying discussion.

**Figure 3:**
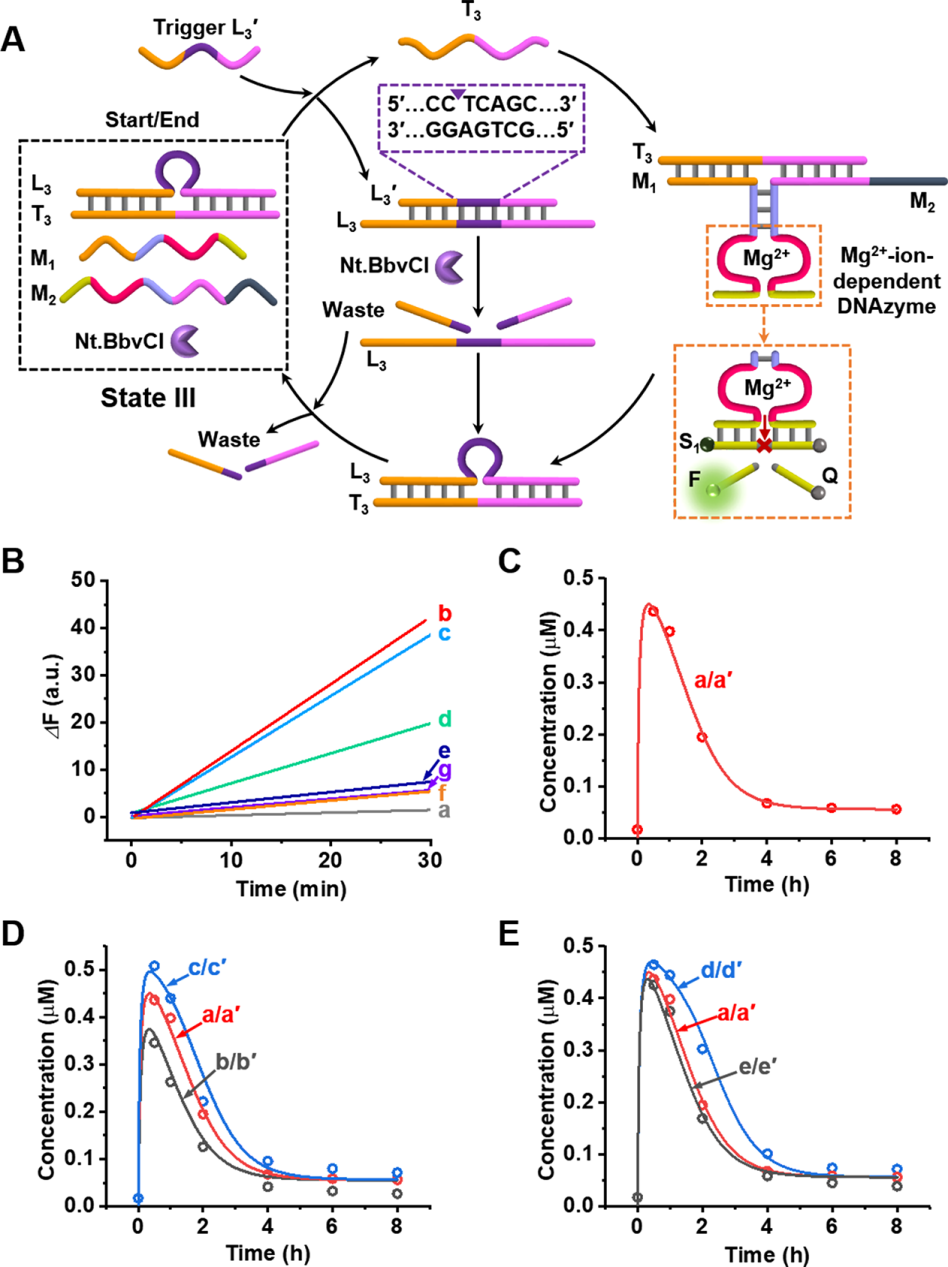
(A) Schematic drawing of the reaction module activating the transient formation and depletion of Mg^2+^-ion-dependent DNAzyme composed of subunit strands. (B) Time-dependent fluorescence changes generated upon the cleavage of S_1_ by Mg^2+^-ion-dependent DNAzyme formed at time-intervals of the transient operation of the reaction module: (a) 0 h, (b) 0.5 h, (c) 1 h, (d) 2 h, (e) 4 h, (f) 6 h, and (g) 8 h. (C) Dots, curve a, transient concentrations of Mg^2+^-ion-dependent DNAzyme; solid line, curve a′, computationally fitted transient concentrations of the DNAzyme upon using the kinetic model of the dynamic reaction module (Figure S13). Experimental and fitted results correspond to the reaction module consisting of: [L_3_/T_3_] = 1 μM, [M_1_] = 1 μM, [M_2_] = 1 μM, [Nt.BbvCI] = 0.046 μM, and [L_3_′] = 5 μM. (D) Characterization of the transient behavior of the reaction module in state III, under different conditions: curves a/a′, experimental and computational results presented in (C); curves b/b′, computationally predicted results (solid line, b′) and experimentally validated results (dots, b) in the presence of [L_3_′] = 3 μM; curves c/c′, computationally predicted results (solid line, c′) and experimentally validated results (dots, c) in the presence of [L_3_′] = 7 μM. All other conditions are as stated in (C). (E) Curves a/a′ are the same as those described in (C); curves d/d′, computationally predicted results (solid line, d′) and experimentally validated results (dots, d) in the presence of [Nt.BbvCI] = 0.023 μM; curves e/e′, computationally predicted results (solid line, e′) and experimentally validated results (dots, e) in the presence of [Nt.BbvCI] = 0.069 μM. All other conditions are the same as those described in (C).

The successful assembly of transient catalytic DNAzymes by reaction modules consisting of appropriately engineered nucleic acid subunits as constituents was then applied to design reaction modules enabling the assembly of other transient DNAzyme nanostructures. Figure 3(A) introduces a reaction module, state III, that allows the transient operation of a Mg^2+^-ion-dependent DNAzyme. The reaction module consists of duplex L_3_/T_3_, the added subunits M_1_, M_2_, and nicking enzyme Nt.BbvCI. Upon triggering the module consisting of state III with L_3_′, the duplex L_3_/T_3_ is displaced to yield the duplex L_3_/L_3_′ and the released strand T_3_, bridges the constituents M_1_/M_2_ to yield Mg^2+^-ion-dependent DNAzyme. Nicking the duplex L_3_/L_3_′ cleaves L_3_′ and the separated strand L_3_ displaces the intermediate Mg^2+^-ion-dependent DNAzyme to yield the energetically stabilized duplex L_3_/T_3_, resulting in the recovery of the rest module in state III. The transient formation and depletion of the intermediate T_3_/M_1_+M_2_ Mg^2+^-ion-dependent DNAzyme was probed by withdrawing samples from the reaction mixture at different time intervals of the dynamic operation of the system, and stimulating Mg^2+^-ion-dependent DNAzyme-induced cleavage of the fluorophore/quencher-modified substrate, S_1_ (F = FAM; Q = BHQ1). The resulting time-dependent fluorescence changes of the cleaved product, Figure 3B reflect the content of the catalytic Mg^2+^-ion-dependent DNAzyme. By applying an appropriate calibration curve corresponding to the rates of the fluorescence changes (λ_em_ = 516 nm) of the cleaved substrate S_1_ by different known standard concentrations of the DNAzyme (Figure S12), the transient concentrations of the formed and dissipated Mg^2+^-ion-dependent DNAzyme, T_3_/M_1_+M_2_ were evaluated, Figure 3C dots. A kinetic model was formulated (Figures S13 and S14 and the accompanying discussion) for the dynamic scheme shown in Figure 3A, and the experimental results were simulated using the kinetic model to yield the best-fit curve, the solid red transient Figure 3C. The set of rate constants derived from the fitted curve are summarized in Table S3. As before, the derived rate constants and kinetic model were used to predict the behavior of the transient system, in the presence of variable concentrations of the trigger, L_3_′ (Figure 3D, curves b′ and c′), and the presence of variable concentrations of the nicking enzyme (Figure 3E, curves d′ and e′). The predicted results were validated experimentally (dots b and c, Figure 3D) and dots d and e, Figure 3E; for the raw experimental curves, see Figure S15). Very good agreement between the computationally predicted transient and the experimental results is demonstrated.

**Figure 4:**
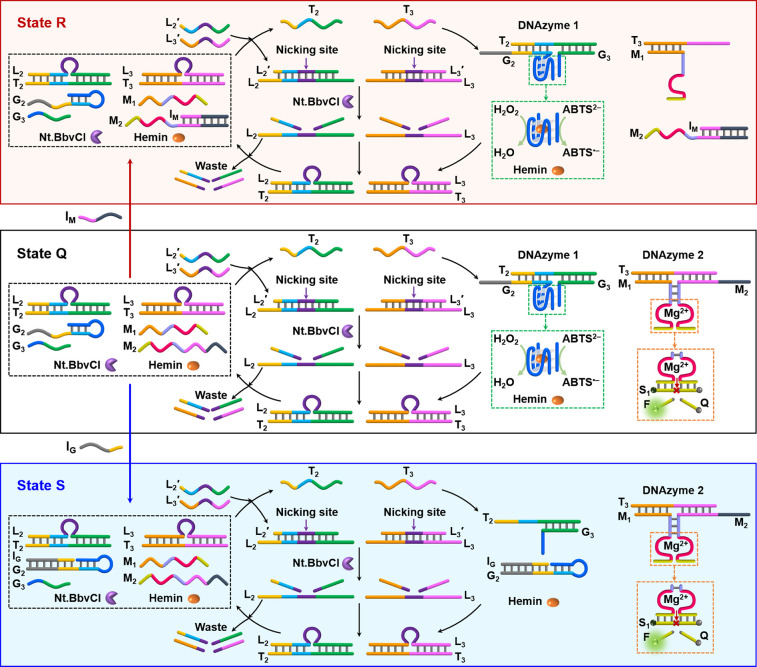
Schematic representation of the gated transient operation of two DNAzymes (DNAzyme 1, the hemin/G-quadruplex; DNAzyme 2, Mg^2+^-ion-dependent DNAzyme). State Q presents the nongated reaction module consisting of the duplexes L_2_/T_2_, L_3_/T_3_, and the constituents G_2_, G_3_, M_1_, and M_2_. Hemin and nicking enzyme Nt.BbvCI are also present. Subjecting state Q to the triggers L_2_′ and L_3_′ leads to the simultaneous transient dynamic formation of DNAzymes 1 and 2. Treatment of state Q with the inhibitor I_M_ blocks the constituent M_2_, leading to the reaction module in state R. Triggering state R with L_2_′ and L_3_′ results in the selective transient formation and depletion of DNAzyme 1 only, while the formation of DNAzyme 2 is blocked. Treatment of state Q with inhibitor I_G_ results in the blockage of the constituent G_2_, leading to the gated transient activation of DNAzyme 2 only, while the formation of DNAzyme 1 is inhibited.

**Figure 5:**
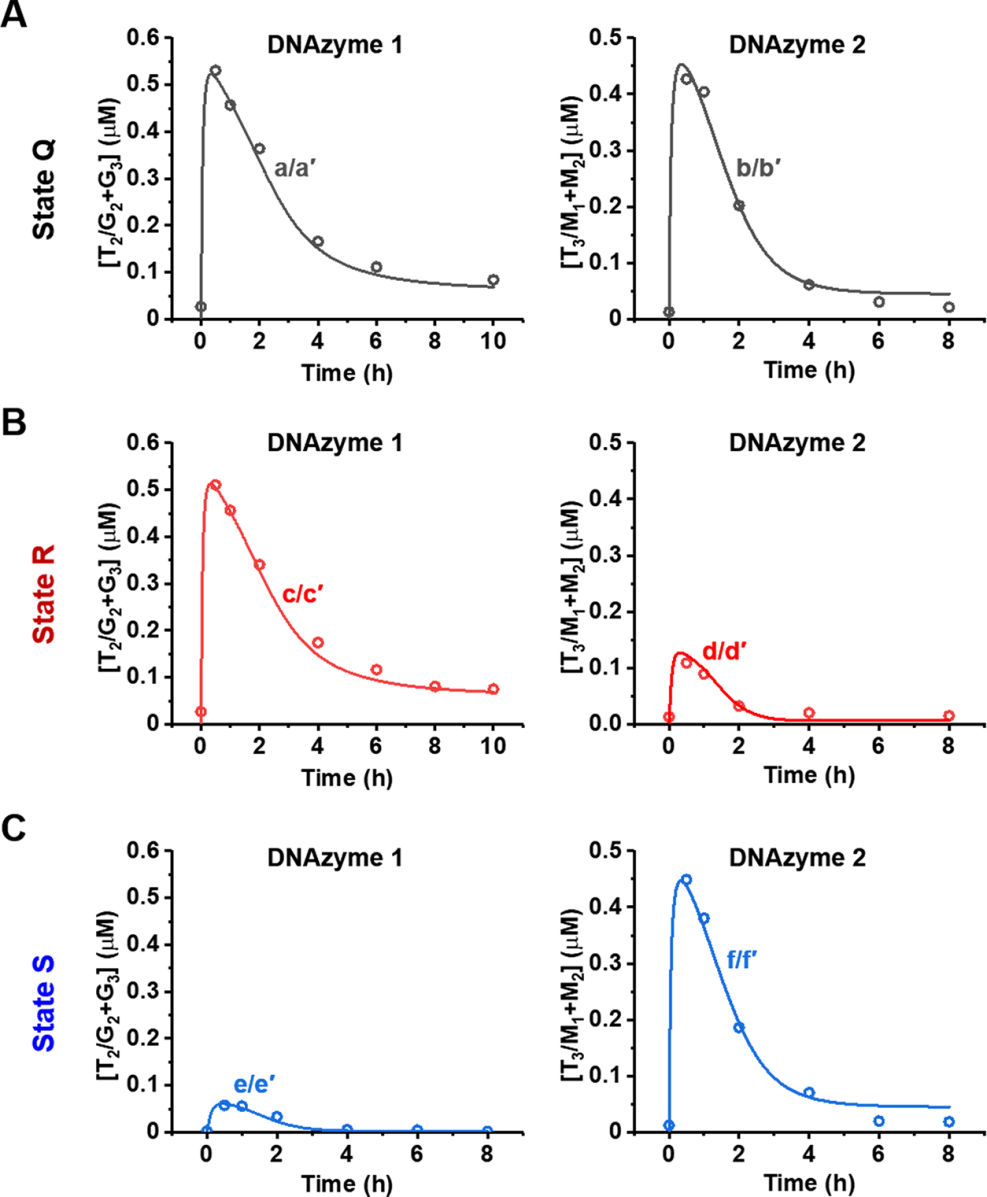
Time-dependent concentration changes of the DNAzyme 1, hemin/G-quadruplex, and DNAzyme 2, Mg^2+^-ion-dependent DNAzyme, upon (A) operation of the nongated reaction mixture in state Q, (B) I_M_ blockage of DNAzyme 2 by the inhibitor-guided transition of state Q to state R and the gated transient operation of DNAzyme 1, and (C) I_G_-induced blockage of DNAzyme 1 by the inhibitor-guided transition of state Q to state S and the gated transient operation of DNAzyme 2. In all curves *i*/*i*′, the transient dots (*i*) correspond to experimental results, and the overlaid solid curves *i*′ correspond to the computationally simulated concentrations of the respective DNAzymes, using the kinetic models formulated in Figures S17–S19. The experimental conditions of the system are as follows: [L_2_/T_2_] = 1 μM, [L_3_/T_3_] = 1 μM, [G_2_] = 1 μM, [G_3_] = 1 μM, [M_1_] = 1 μM, [M_2_] = 1 μM, [hemin] = 1 μM, [Nt.BbvCI] = 0.069 μM, [L_2_′] = 4 μM, [L_3_′] = 5 μM, [I_M_] = 2 μM (only state R), and [I_G_] = 2 μM (only state S).

The successful design of two reaction modules that guide the transient operation of two different DNAzymes was then applied to develop the gated operation of the two DNAzymes, Figure 4. The system, state Q, consists of a mixture of the two duplexes L_2_/T_2_ and L_3_/T_3_, the G-quadruplex subunit constituents G_2_ and G_3_, strands M_1_ and M_2_, and the nicking enzyme catalyst, Nt.BbvCI. Subjecting this composite to triggers L_2_′ and L_3_′ simultaneously leads to the parallel, nongated, transient operation of the hemin/G-quadruplex DNAzyme, catalyzing the oxidation of ABTS^2–^ to ABTS^•–^ by H_2_O_2_ and the cleavage of the substrate S_1_, generating the fluorescence changes. The parallel transient operation of the hemin/G-quadruplex DNAzyme, T_2_/G_2_+G_3,_ and of Mg^2+^-ion-dependent DNAzyme, T_3_/M_1_+M_2_, are presented in Figure 5A (the time-dependent catalytic curves of the two DNAzymes at time intervals are shown in Figure S16). A kinetic model combing the two transient DNAzymes was formulated (Figure S17) for state Q. Using the set of rate constants (summarized in Table S4) which were derived from the fitted curves of the individual transient DNAzymes, Figure 2 and 3, the transient dissipative curves corresponding to the parallel nongated DNAzymes were predicted, curve a′ and b′, and these are in good agreement with the experimental results. In order to achieve gated operation of the system, strand M_2_ was pre-engineered to include a toehold domain that could hybridize with inhibitor strand I_M_. Treatment of the system in state Q with inhibitor I_M_ therefore yields the reaction module in state R where strand M_2_ is blocked. The L_2_′- and L_3_′-triggered separation of duplexes L_2_/T_2_ and L_3_/T_3_ leads to duplexes L_2_/L_2_′ and L_3_/L_3_′ and to separated strands T_2_ and T_3_. While T_2_ results in the formation of hemin/G-quadruplex DNAzyme as before, the T_3_-stimulated formation of Mg^2+^-ion-dependent DNAzyme is inhibited by the blocking strand. In turn, the nicking of strands L_2_′ and L_3_′ in duplexes L_2_/L_2_′ and L_3_/L_3_′, by Nt.BbvCI leads to the formation of free L_2_, L_3_ that rehybridize with T_2_, T_3_ to recover to the rest reaction module, state R. Thus, subjecting state R to triggers L_2_′ and L_3_′ leads to the gated activation of hemin/G-quadruplex DNAzyme, yet the operation of the Mg^2+^-ion-dependent DNAzyme does not occur. Figure 5B shows that in the presence of I_M_ the gated transient operation of the hemin/G-quadruplex DNAzyme, T_2_/G_2_+G_3_, proceeds effectively, while the activity of Mg^2+^-ion-dependent DNAzyme, T_3_/M_1_+M_2_, is almost fully blocked. Similarly, treatment of the mixture in state Q with the inhibitor strand I_G_ (pre-engineered to block strand G_2_) results in the hybridization of I_G_ with the single strand toehold sequence engineered into hairpin G_2_, yielding the reaction module in state S. Interacting the system in state S with the two triggers L_2_′ and L_3_′ leads to the gated operation of the transient Mg^2+^-ion-dependent DNAzyme, while the formation of the hemin/G-quadruplex DNAzyme is inhibited, since T_2_ can not unlock blocked hairpin structure G_2_. Figure 5C demonstrates that the activity of the hemin/G-quadruplex is almost fully blocked, while the transient activity of Mg^2+^-ion-dependent DNAzyme is switched on. Two kinetic models that account for the transient gated operation of the two DNAzymes in the presence of inhibitors I_M_ and I_G_ were respectively formulated (Figures S18 and S19). As these models include a set of rate constants that are involved in the transient operation of the individual DNAzymes, that were computationally derived and experimentally supported, we adopted this set of rate constants and integrated them into the comprehensive kinetic model of the gated DNAzymes that include all rate constants associated with the participation of the inhibitors in the dynamic process. The kinetic models were applied computationally to the experimental results of the gated transient operation of the two DNAzymes (solid curves overlaid on the experimental dots, Figure 5). The sets of rate constants corresponding to the set of reactions associated with the kinetic models are summarized in Tables S5 and S6. These sets of rate constants were used to predict the performance of the gated DNAzyme systems at auxiliary conditions that differ from those applied to derive Figure 5. The predicted results at different auxiliary conditions and the experimental validation of the predicted results, are presented in Figures S20 and S21 and the accompanying discussion.

**Figure 6:**
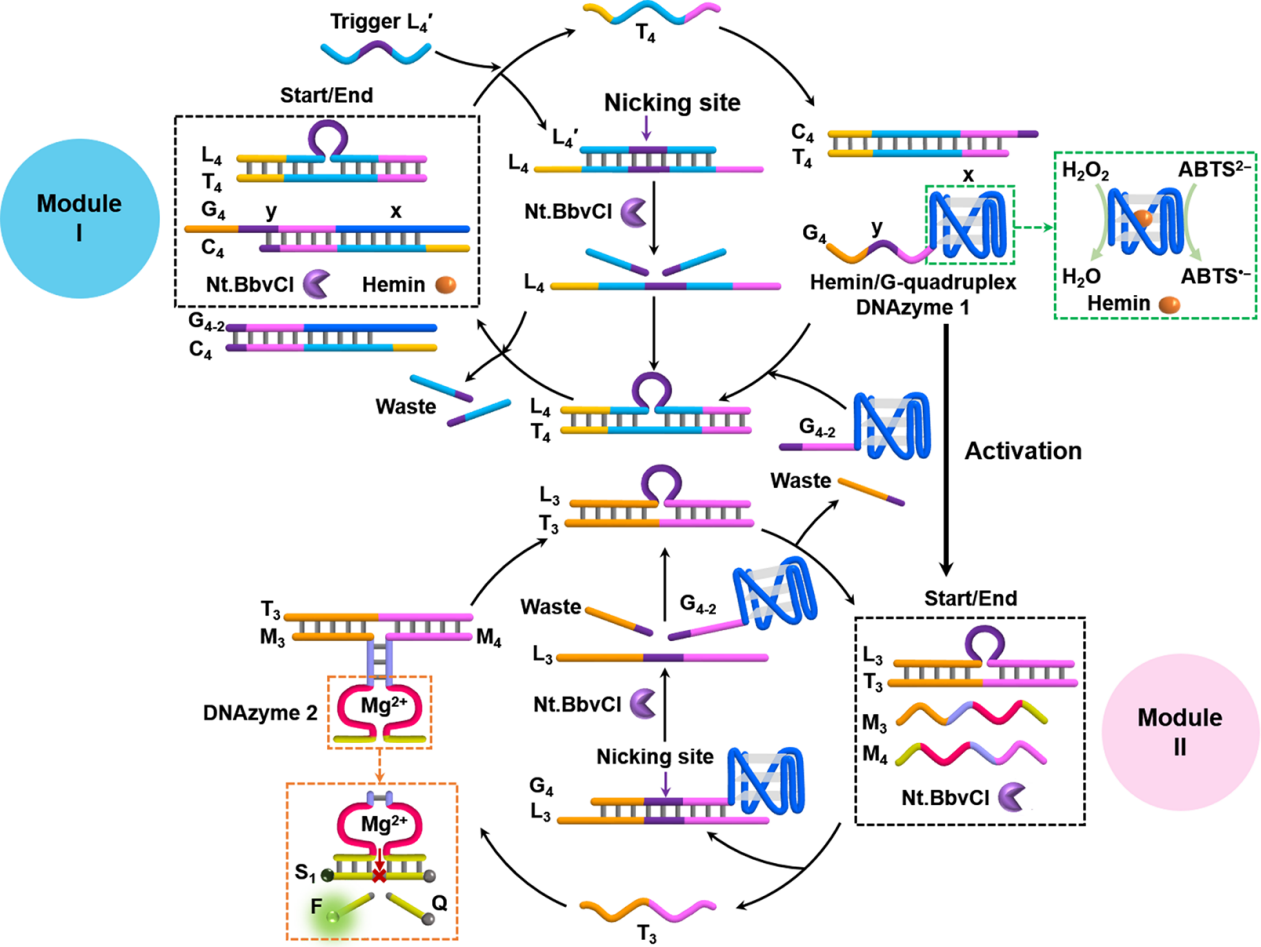
Schematic illustration of the assembly of a dynamic transient cascade of two DNAzymes (DNAzyme 1, hemin/G-quadruplex, and DNAzyme 2, Mg^2+^-ion-dependent DNAzyme). The cascaded process is activated by intercommunication of two transient operating modules: modules I and II.

**Figure 7:**
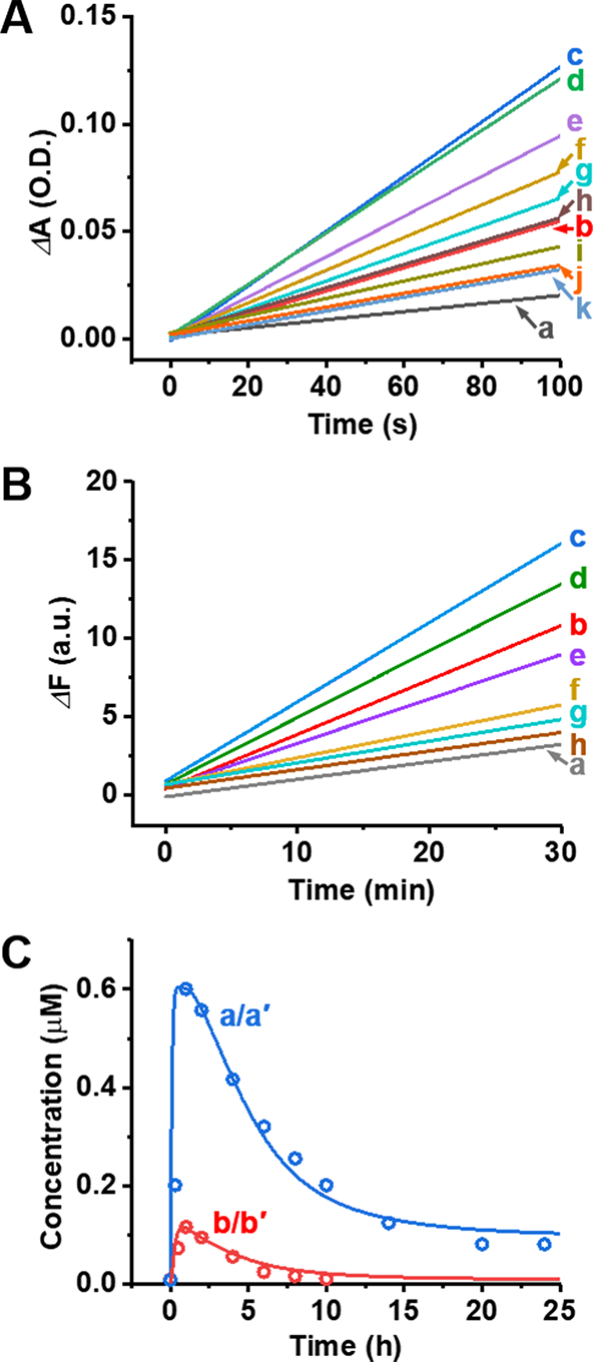
(A) Time-dependent absorbance changes generated by DNAzyme 1, the hemin/G-quadruplex, at time intervals of operation of the two-enzyme cascade: (a) 0 h, (b) 0.3 h, (c) 1 h, (d) 2 h, (e) 4 h, (f) 6 h, (g) 8 h, (h) 10 h, (i) 14 h, (j) 20 h, and (k) 24 h. (B) Time-dependent fluorescence changes generated by Mg^2+^-ion-dependent DNAzyme formed in the system at different time-intervals of operation of the dynamic transient biocatalytic cascade: (a) 0 h, (b) 0.5 h, (c) 1 h, (d) 2 h, (e) 4 h, (f) 6 h, (g) 8 h, and (h) 10 h. (C) Transient concentrations corresponding to DNAzyme 1, curve a, transient dots; DNAzyme 2, curve b, transient dots. The overlaid solid lines (curves a′ and b′) correspond to the computationally simulated results using the kinetic model formulated in Figure S23, and the resulting rate constants are summarized in Table S7. The transient concentrations of the respective DNAzyme were evaluated from the time-dependent absorbance/fluorescence changes generated by the two DNAzymes on applying the respective calibration curves. The reaction modules used for the cascaded DNAzyme process included [L_4_/T_4_] = 2 μM, [G_4_/C_4_] = 2 μM, [hemin] = 2 μM, [L_3_/T_3_] = 1 μM, [M_3_] = 1 μM, [M_4_] = 1 μM, [Nt.BbvCI] = 0.069 μM, [L_4_′] = 6 μM.

Besides the gated operation of the two transient DNAzymes, the cascaded operation of the two DNAzymes was achieved. Figure 6 depicts the scheme developed to intercommunicate between two reaction modules that allows the Nt.BbvCI-catalyzed operation of the DNAzyme cascade consisting of the hemin/G-quadruplex and Mg^2+^-ion-dependent DNAzymes. The system is composed of two reaction modules: modules I and II. Module I includes duplexes L_4_/T_4_ and G_4_/C_4_ and nicking enzyme Nt.BbvCI in its rest state. Module II includes duplex L_3_/T_3_, the subunits M_3_ and M_4_ and nicking enzyme Nt.BbvCI in its rest state. The triggered activation of module I by L_4_′ displaces duplex L_4_/T_4_ to yield L_4_/L_4_′, and the released T_4_ displaces duplex G_4_/C_4_ to yield duplex T_4_/C_4_ and to release strand G_4_ that self-assembles into G-quadruplex-based DNAzyme 1. The nicking of strand L_4_′ in duplex L_4_/L_4_′ separate L_4_ that displaces intermediate duplex T_4_/C_4_ to yield energetically stabilized L_4_/T_4_, and the released C_4_ acts as functional unit to separate G-quadruplex and regenerate the reaction module. That is, the L_4_′-triggered dynamic operation of module I includes the machinery necessary to regenerate the rest of module I. Concomitant to this transient dynamic path, the transiently formed G-quadruplex product includes, however, the encoded information, i.e., extended single-strand tether *y* that acts as functional unit, to interact with module II and to activate the transient cascaded DNAzyme. In parallel to the dynamic process proceeding in module I, tether *y*, associated with the G-quadruplex, displaces duplex L_3_/T_3_ associated with module II to yield duplex G_4_/L_3_ and free strand T_3_. The released strand T_3_ bridges subunits M_3_ and M_4_ to self-assemble the supramolecular Mg^2+^-ion-dependent DNAzyme that cleaves the fluorophore-quencher-modified substrate, S_1_. Tether *y* of G_4_, hybridized in duplex G_4_/L_3_, includes, however, the sequence to be nicked by Nt.BbvCI, and the cleavage of G_4_ yields fragment G_4–2_ and releases strand L_3_. The released strand L_3_ displaces strand T_3_ associated with Mg^2+^-ion-dependent DNAzyme, resulting in the dynamic transient separation of Mg^2+^-ion-dependent DNAzyme 2 and the regeneration of the rest state of module II. The released “waste” strand product, G_4–2_, generated upon the cleavage of duplex G_4_/L_3_ includes, in its free tether, the engineered sequence *x* that includes the capacity to displace intermediate duplex T_4_/C_4_ of module I to yield duplex G_4–2_/C_4_, thereby cooperatively assisting the transient recovery of the rest of module I by depleting the intermediate, dynamically generated component formed in module I. That is, the L_4_′-triggered coupling of modules I and II leads to the dynamic transient cascaded activation of two DNAzymes: the hemin/G-quadruplex DNAzyme (in module I) and Mg^2+^-ion-dependent DNAzyme (in module II). The catalytic oxidation of ABTS^2–^ to ABTS^•–^ (λ = 420 nm) by the hemin/G-quadruplex DNAzyme, at different time-intervals during the operation of the transient cascades (Figure 7A) and the time-dependent fluorescence changes associated with the cleavage of the substrate S_1_ by Mg^2+^-ion-dependent DNAzyme, at different time-intervals of the transient catalytic cascade (Figure 7B), provide readout signals for the biocatalytic cascade. Using the appropriate calibration curves (Figure S22), the transient concentrations corresponding to the formation and depletion of the catalytic DNAzymes 1 and 2 were evaluated (Figure 7C, dots, curves a and b). A kinetic model that accounts for the transient cascaded DNAzyme system was formulated (Figure S23). This kinetic model includes a set of rate constants that are associated with the individual DNAzyme previously computationally and experimentally supported and add rate constants associated with the dynamic communication between the two cascaded reaction modules (Table S7). The integrated kinetic model was then applied to computationally fit the experimental transients of the DNAzymes participating in the two-enzymes cascade. The computational transient curves are overlaid on the experimental results (solid line curves a′ and b′, Figure 7C).

## Conclusion

The study introduced synthetic systems driving the transient operation of G-quadruplexes, a dynamic gated system of transient DNAzymes, and a transient cascaded system of DNAzymes. Realizing the significance of dynamic formation and depletion of G-quadruplexes in controlling the replication and transcription of genes and the consequences of malfunctions of this process in causing diseases, the present study introduces synthetic modules to form and separate G-quadruplexes. In fact, substantial efforts are directed to develop methods to form and unwind G-quadruplexes^76,77^ as therapeutic means to fight G-quadruplex related diseases. Metal ions,^78^ complexes,^79,80^ photoresponsive ligands,^81,82^ and natural polyamines such as spermine^83^ were used to control G-quadruplexes stability and topology. In this context, coupling functional DNA machineries to G-quadruplex structures as a means to dissipatively perturb G-quadruplex structures could provide a versatile path for the spatiotemporal regulation of G-quadruplexes. The different systems described in the study operated, however, in homogeneous buffer solutions. As future applications of such networks are envisaged for therapeutic applications, the use of the artificial networks in native bioenvironments is an important goal. Indeed, G-quadruplexes and Mg^2+^-ion-dependent DNAzymes were suggested as catalytic agents for cancer therapy.^84−86^ Thus, toward the possible use of such artificial networks in native media, we examined the operation of the transient network displayed in Figure 2 in a cancer cell lysate. The results and the accompanying discussion are presented in Figure S24. We find that the transient system shown in Figure 2 successfully operates in the cell lysate, yet the cell lysate affects the dynamics of the transient process as compared to the pure buffer solution. We find that the activity of nicking enzyme Nt.BbvCI is lowered by ca. 25% in the cell lysate, and this results in a slightly elevated peak content of transient complex T_2_/G_2_+G_3_ and a slower recovery of the parent reaction module. Nonetheless, the results indicate the feasibility to operate such artificial networks in native environments.

## Experimental Section

### Oligonucleotides

Oligonucleotides were purchased from Sigma-Aldrich and Integrated DNA Technologies, Inc. The following sequence strands (5′ → 3′) were used to construct the different systems: (1) G_1_, TTTGGGTAGGGCGGGTTGGG; (2) C_1_: CATCAATCCCAACCCGTCCTACC; (3) T_1_, TTTTTTTTTTATAGGACGGGTTAGGATTGAT; (4) L_1_, TTTTTTTTTCAATCCTA***GCTGAGG***CCCGTCCTATA; (5) L_1_′, CGGG***CCTCAGC***TAG; (6) G_2_, TACAGCTCCTAGTTTAGCCGCCATGGGTAGGGCGGG; (7) G_3_, TTGGGTAGAGATGCTGC; (8) T_2_, GCAGCATCTCTTGGCGGCTAAACT; (9) L_2_, AGTTTAGCC***GCTGAGG***AAGAGATGCTGC; (10) L_2_′, TCTT***CCTCAGC***GGCT; (11) I_G_, CTAAACTAGGAGCTGTA; (12) S_1_, FAM-ACTGAAT**rA**GGCTGGA-BHQ1; (13) M_1_, ACAGAAGAACCGGCTG*CACCCATGTTTCAGT*; (14) M_2_, *TCCAGCAGCGAT*CAGCCGCCATCACAAAGGAAAGAGGCAGG; (15) T_3_, GGTTTGTGATGGACGTTCTTCTGTC; (16) L_3_, GACAGAAGAAC***GCTGAGG***CCATCACAAACC; (17) L_3_′, GATGG***CCTCAGC***GTT; (18) I_M_, CCTGCCTCTTTCCTTTG; (19) Cy3-labeled L_3_, Cy3-GACAGAAGAAC***GCTGAGG***CCATCACAAACC; (20) BHQ2-labeled L_3_′, GATGG***CCTCAGC***GTT-BHQ2; (21) G_4_, GATGGCCTCAGCGTTTGGGTAGGGCGGGTTGGG; (22) C_4_, CATGTTCCCGCCCTTTCCAAACGCT; (23) T_4_, GTTTGGAATGGGCAGGAACATGT; (24) L_4_, ACATGTTCCTG***GCTGAGG***CCCATTCCAAA; (25) L_4_′, TGGG***CCTCAGC***CAGG; (26) M_3_, ACAGAAGAACCGGCTG*CACCCATGTTTCAGT*; (27) M_4_, *TCCAGCAGCGAT*CAGCCGCCATCACAAA. The sequences for folding into G-quadruplex are underlined. Mg^2+^-ion-dependent DNAzyme sequences are underlined in italics. The ribonucleobase cleavage site, rA, in the substrate S_1_ is presented in bold. The sequence domains recognized by Nt.BbvCI are indicated in bold italics.

### Preparation and Measurement of Transient, Dissipative DNAzyme Systems

The composition and characterization of the different systems discussed in the paper are detailed in the Supporting Information. As an example, the composition and operation of the cascaded system presented in Figures 6 and 7 are described in brief below, and further details are presented in the Supporting Information. The cascaded system consisted of a mixture of T_4_/L_4_ (2 μM), G_4_/C_4_ (2 μM), T_3_/L_3_ (1 μM), M_3_ (1 μM), M_4_ (1 μM), Nt.BbvCI (0.069 μM), and hemin (2 μM) in 1× rCutSmart Buffer, with a total volume of 1.0 mL. Upon adding trigger L_4_′ (6 μM) to the mixture (0 h time point), the dissipative system was incubated at 33 °C. For the measurement of the time-dependent absorbance changes of ABTS^•–^ catalyzed by hemin/G-quadruplex DNAzyme in the cascaded system, 40 μL aliquots of the mixture were withdrawn at different time intervals. Each aliquot was mixed with 20 μL of ABTS^2–^ (1 mM) and 20 μL of H_2_O_2_ (1 mM) in a quartz cuvette with 10 mm path length. The time-dependent absorbance changes of ABTS^•–^ at 420 nm (ε = 36 000/M·cm) were recorded at 25 °C on a UV-2450 spectrophotometer (Shimadzu). For the measurement of the time-dependent fluorescence changes of the cleavage of S_1_ catalyzed by Mg^2+^-ion-dependent DNAzyme in the cascaded system, aliquots of 50 μL were withdrawn at different time intervals and treated with 50 μL of S_1_ (2 μM, supplemented with 10 mM Mg^2+^). The time-dependent fluorescence changes (λ_ex_ = 496 nm, λ_em_ = 516 nm) of S_1_ were monitored using a plastic cuvette with 10 mm path length at 25 °C on a Cary Eclipse Fluorometer (Varian, Inc.).

## Supporting Information Available

Chemicals, preparation of different transient DNAzyme systems, measurement methods, characterizations, calibration curves corresponding to the analyses of different systems, CD spectra, gel electrophoresis, time-dependent absorbance changes of ABTS^•–^, absorbance spectra of ABTS^•–^, fluorescence changes of the cleaved S_1_ at different time intervals of transient DNAzyme systems, computational kinetic models for simulations, tables summarizing the rate constants corresponding to different systems, cell lysate experiment (PDF)

## Dedication

In memory of Prof. Nadrian C. Seeman, a pioneer in DNA nanotechnology.
