# Supplementary material for: Assembly of Dynamic Gated and Cascaded Transient DNAzyme Networks
Source: ACS Nano. 2022 Mar 16;16(4):6153–64. doi: 10.1021/acsnano.1c11631 (PMC9047661; doi:10.1021/acsnano.1c11631)
Supplement: Supplementary file 1 — nn1c11631_si_001.pdf [file nn1c11631_si_001.pdf]

Supporting Information

Assembly of Dynamic Gated and Cascaded Transient DNAzyme Networks

*Jiantong Dong, Yu Ouyang, Jianbang Wang, Michael P. O'Hagan, Itamar Willner**

Institute of Chemistry, Center for Nanoscience and Nanotechnology, The Hebrew University of Jerusalem, Jerusalem, 91904, Israel.

*Corresponding author email: itamar.willner@mail.huji.ac.il

Chemicals

2,2'-Azino-bis(3-ethylbenzothiazoline-6-sulfonic acid) ($ABTS^{2-}$), hydrogen peroxide, hemin, potassium chloride, magnesium chloride, acrylamide/bis-acrylamide (40% solution, 19:1), ammonium persulfate, *N,N,N',N'*-tetramethylethylenediamine, and DNA oligonucleotides were purchased from Sigma-Aldrich. Nicking endonuclease Nt.BbvCI (10,000 units/mL, 2.3 μM), and 10 × rCutSmart Buffer (50 mM potassium acetate, 10 mM tris-acetate, 10 mM magnesium acetate, 100 μg/mL recombinant albumin, pH 7.9 @ 25 °C) were purchased from New England BioLabs Inc. GelRed nucleic acid gel stain (10,000 × in DMSO) was purchased from Biotium. 5 × TBE electrophoresis buffer was purchased from Biological Industries. The fluorophore and quencher-labeled DNA probes were purchased from Integrated DNA Technologies Inc.

Preparation of the transient dissipative DNAzyme systems

To prepare the transient dissipative assembly of hemin/G-quadruplex DNAzyme shown in Figure 1, T_1/L_1 and G_1/C_1, 10 μM each, in 1 × rCutSmart Buffer, were annealed at 90 °C for 5 min and cooled down to 25 °C over 1 h. A mixture of T_1/L_1, G_1/C_1 and hemin, 1 μM each, in 1 × rCutSmart Buffer, were subjected to variable concentrations of Nt.BbvCI and L_1'. The mixture, with a total volume of 0.4 mL, was incubated at 33 °C.

To prepare the transient dissipative assembly of supramolecular hemin/G-quadruplex DNAzyme shown in Figure 2, T_2/L_2, G_2, 10 μM each, in 1 × rCutSmart Buffer, were annealed at 90 °C for 5 min and cooled down to 25 °C over 1 h. A mixture of T_2/L_2, G_2, G_3 and hemin, 1 μM each, in 1 × rCutSmart Buffer, were subjected to variable concentrations of Nt.BbvCI and L_2'. The mixture, with a total volume of 0.4 mL, was incubated at 33 °C.

For the transient dissipative assembly of Mg^{2+}-ion-dependent DNAzyme shown in Figure 3, 10 μM of T_3/L_3 in 1 × rCutSmart Buffer were annealed at 90 °C for 5 min and cooled down to 25 °C over 1 h. A mixture of T_3/L_3, M_1 and M_2, 1 μM each, in 1 × rCutSmart Buffer, were subjected to the variable concentrations of Nt.BbvCI and L_3'. The mixture, with a total volume of 0.4 mL, was incubated at 33 °C.

For the gated transient assembly of DNAzymes (State Q, non-gated) shown in Figure 4, a mixture of T_2/L_2, T_3/L_3, G_2, G_3, M_1, M_2 and hemin, 1 μM each, in 1 × rCutSmart Buffer, were subjected to 0.069 μM of Nt.BbvCI, 4 μM of L_2', and 5 μM of L_3'. For the inhibitor I_M-gated transient assembly of DNAzymes (State R), a mixture of T_2/L_2, T_3/L_3, G_2, G_3, M_1, M_2 and hemin, 1 μM each, in 1 × rCutSmart Buffer, were subjected to different concentrations of inhibitor I_M (1 or 2 μM), 0.069 μM of Nt.BbvCI, 4 μM of L_2' and 5 μM of L_3'. For the inhibitor I_G-gated transient assembly of DNAzymes (State S), a mixture of T_2/L_2, T_3/L_3, G_2, G_3, M_1, M_2 and hemin, 1 μM each, in 1 × rCutSmart Buffer, were subjected to different concentrations of inhibitor I_G (1 or 2 μM), 0.069 μM of Nt.BbvCI, 4 μM of L_2' and 5 μM of L_3'. For each system, the mixture, with a total volume of 0.8 mL, was incubated at 33 °C.

For the cascaded transient dynamic assembly of DNAzymes shown in Figure 6, a mixture of T_4/L_4 (2 μM), G_4/C_4 (2 μM), hemin (2 μM), T_3/L_3 (1 μM), M_3 (1 μM), M_4 (1 μM), in 1 × rCutSmart Buffer, were subjected to 0.069 μM of Nt.BbvCI and 6 μM of L_4'. The mixture, with a total volume of 1.0 mL, was incubated at 33 °C.

Measurement of the absorbance changes of $ABTS^{\bullet-}$ catalyzed by the transiently assembled hemin/G-quadruplex DNAzyme

For each dissipative system prepared as described above, aliquots of 40 μL of the mixture were withdrawn from the transiently assembled DNAzyme systems at different time intervals. $ABTS^{2-}$ and H_2O_2 were added to the mixture to yield a final volume of 80 μL (for the G_1 and T_2/G_2+G_3 -assembled hemin/G-quadruplex DNAzyme, the final concentration of $ABTS^{2-}$ was 0.25 mM and H_2O_2 was 0.50 mM; for G_4-assembled hemin/G-quadruplex DNAzyme, the final concentration of $ABTS^{2-}$ and H_2O_2 were both 0.25 mM). The time-dependent absorbance changes of $ABTS^{\bullet-}$ at 420 nm (ε = 36,000 /(M·cm)) by the transient assembled hemin/G-quadruplex DNAzyme were recorded using a quartz cuvette with 10-mm path length at 25 °C on a UV-2450 spectrophotometer (Shimadzu). The catalytic rate (μM/min) was calculated as $d(\Delta A/0.036)/d(t/60)$. By using the calibration curves derived from measuring the catalytic rates of standard concentrations of hemin/G-quadruplex DNAzyme, the

concentrations of the transiently assembled hemin/G-quadruplex DNAzyme at time intervals were determined.

Measurement of the fluorescence changes of the substrate catalyzed by the transiently assembled Mg^{2+}-ion-dependent DNAzyme

For each dissipative system prepared, aliquots of 50 μL were withdrawn at time intervals and treated with 50 μL of S_1 stock solution (2 μM, supplemented with 10 mM Mg^{2+}). The time-dependent fluorescence changes (λ_{ex} = 496 nm, λ_{em} = 516 nm) generated by the cleavage of S_1 were monitored using a plastic cuvette with 10-mm path length at 25 °C on a Cary Eclipse Fluorometer (Varian Inc). By using the calibration curves derived from measuring the catalytic rate (d(ΔF)/dt) by standard concentrations of the Mg^{2+}-ion-dependent DNAzyme, the concentrations of the transient Mg^{2+}-ion-dependent DNAzyme were quantified.

Circular dichroism spectroscopy

For the measurement of circular dichroism (CD) spectra of the G-quadruplex in the transient dissipative system, samples (each 120 μL) were prepared in 1 × rCutSmart buffer. CD spectra were recorded using a quartz cuvette with 10-mm path length at room temperature on a CD spectrometer (MOS-500, Bio-Logic Science Instruments).

Gel electrophoresis

For each dissipative system prepared, aliquots of 10 μL were withdrawn and incubated with 1 μL of trypsin (2 mg/mL) for 5 min to inactivate the nicking enzyme Nt.BbvCI, and stop the dissipative process at the desired time intervals. Native polyacrylamide gel electrophoresis experiments were performed using polyacrylamide gel (12%, 19:1 acrylamide/bis-acrylamide) with a gel thickness of 1.0 mm, and run using a Hoefer SE 600 electrophoresis unit at 80 V and at 5 °C for 18 h. Following electrophoresis, DNA strands were visualized by staining with 1 × GelRed and imaged using a Fusion FX-Vilber Lumart instrument. The intensity of the bands was quantitatively evaluated by Image J software.

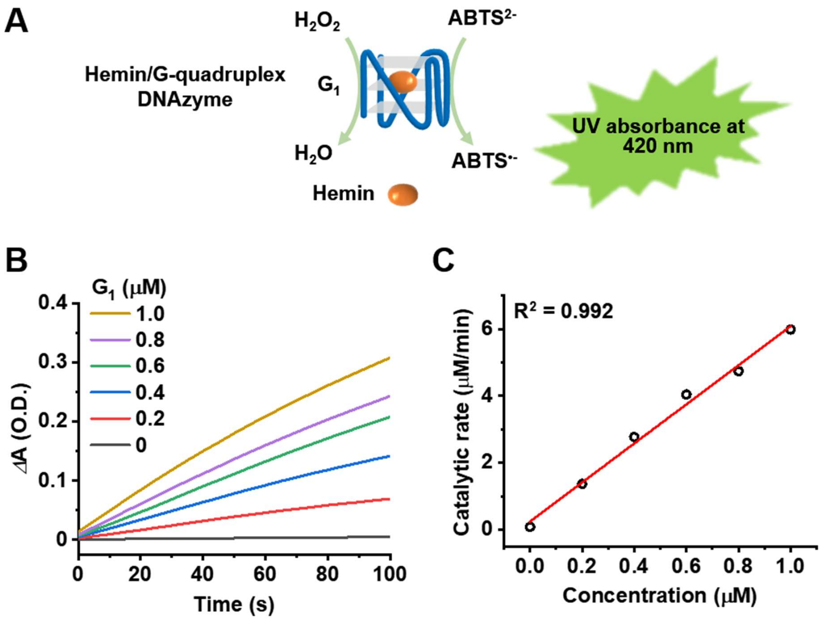

Figure S1. (A) Schematic illustration of the oxidation of $ABTS^{2-}$ to $ABTS^{\bullet-}$ by the hemin/G-quadruplex DNAzyme in the presence of H_2O_2. (B) The time-dependent absorbance changes of $ABTS^{\bullet-}$ upon the addition of variable concentrations of G_1 to form the hemin/G-quadruplex DNAzyme. (C) The derived calibration curve corresponding to the catalytic rate of $ABTS^{\bullet-}$ at different standard concentrations of G_1-assembled hemin/G-quadruplex DNAzyme. $ABTS^{2-}$ concentration was 0.25 mM and H_2O_2 concentration was 0.50 mM.

Computational kinetic simulations of the experimental results

Kinetic models for each of the transient systems described in the study were formulated. The initial concentrations of the constituents used in the simulations of the different systems are provided for each of the models. The simulation process employs the law of mass conservation of the intermediates as a prerequisite. The set of rate constants associated with the optimized fitted curves were evaluated. To support the derived set of rate constants as a meaningful representative solution for the system (rather than a coincidental local solution), we used this set of rate constants to predict the behavior of the system at different auxiliary conditions and validated the predicted results by appropriate experiments. When possible, the set of simulated rate constants was supplemented by independent evaluation of experimental rate constants. For example, the simulated rate constants for the transient system displayed in Figure 3 and the accompanying kinetic model in Figure S13 were validated by the experimental measurement of rate constants k_{15} and k_{-15} (Figure S14). The experimental rate constants are in good agreement with the simulated values. In fact, these two rate constants are also a part of the system presented in Figure 4 and Figure 5. The simulated rate constants of the later system were adjusted to fit the experimental results, so that the resulting rate constants present a global set of values. The tables of rate constants included in the Supporting Information represent the set of rate constants associated with the respective systems, and the values noted below the tables correspond to experimentally validated values.

Kinetic equations of the transient dissipative hemin/G-quadruplex DNAzyme system shown in Figure 1:

(1) $L_1 + T_1 \underset{k_{-1}}{\overset{k_1}{\rightleftarrows}} L_1T_1$

(2) $G_1 + C_1 \underset{k_{-2}}{\overset{k_2}{\rightleftarrows}} G_1C_1$

(3) $L_1T_1 + L_1' \underset{k_{-3}}{\overset{k_3}{\rightleftarrows}} L_1L_1' + T_1$

(4) $G_1C_1 + T_1 \underset{k_{-4}}{\overset{k_4}{\rightleftarrows}} T_1C_1 + G_1$

(5) $L_1L_1' + E \underset{k_{-5}}{\overset{k_5}{\rightleftarrows}} L_1L_1'E$

(6) $L_1L_1'E \xrightarrow{k_6} E + L_1L_{1-1}'L_{1-2}'$

(7) $L_1L_{1-1}'L_{1-2}' \underset{k_{-7}}{\overset{k_7}{\rightleftarrows}} L_1 + L_{1-1}' + L_{1-2}'$

Derivatives:

$$\frac{dL_1}{dt} = k_{-1}[L_1T_1] - k_1[L_1][T_1] + k_7[L_1L_{1-1}'L_{1-2}'] - k_{-7}[L_1][L_{1-1}'][L_{1-2}']$$

$$\frac{dT_1}{dt} = k_{-1}[L_1T_1] - k_1[L_1][T_1] + k_3[L_1T_1][L_1'] - k_{-3}[L_1L_1'][T_1] + k_{-4}[T_1C_1][G_1] - k_4[G_1C_1][T_1]$$

$$\frac{dL_1T_1}{dt} = k_1[L_1][T_1] - k_{-1}[L_1T_1] + k_{-3}[L_1L_1'][T_1] - k_3[L_1T_1][L_1']$$

$$\frac{dG_1}{dt} = k_{-2}[G_1C_1] - k_2[G_1][C_1] + k_4[G_1C_1][T_1] - k_{-4}[T_1C_1][G_1]$$

$$\frac{dC_1}{dt} = k_{-2}[G_1C_1] - k_2[G_1][C_1]$$

$$\frac{dG_1C_1}{dt} = k_2[G_1][C_1] - k_{-2}[G_1C_1] + k_{-4}[T_1C_1][G_1] - k_4[G_1C_1][T_1]$$

$$\frac{dL_1'}{dt} = k_{-3}[L_1L_1'][T_1] - k_3[L_1T_1][L_1']$$

$$\frac{dL_1L_1'}{dt} = k_3[L_1T_1][L_1'] - k_{-3}[L_1L_1'][T_1] + k_{-5}[L_1L_1'E] - k_5[L_1L_1'][E]$$

$$\frac{dT_1C_1}{dt} = k_4[G_1C_1][T_1] - k_{-4}[T_1C_1][G_1]$$

$$\frac{dE}{dt} = k_{-5}[L_1L_1'E] - k_5[L_1L_1'][E] + k_6[L_1L_1'E]$$

$$\frac{dL_1L_1'E}{dt} = k_5[L_1L_1'][E] - k_{-5}[L_1L_1'E] - k_6[L_1L_1'E]$$

$$\frac{dL_1L_{1-1}'L_{1-2}'}{dt} = k_6[L_1L_1'E] + k_{-7}[L_1][L_{1-1}'][L_{1-2}'] - k_7[L_1L_{1-1}'L_{1-2}']$$

$$\frac{dL_{1-1}'}{dt} = k_7[L_1L_{1-1}'L_{1-2}'] - k_{-7}[L_1][L_{1-1}'][L_{1-2}']$$

$$\frac{dL_{1-2}'}{dt} = k_7[L_1L_{1-1}'L_{1-2}'] - k_{-7}[L_1][L_{1-1}'][L_{1-2}']$$

Figure S2. Computational simulation of the transient dissipative hemin/G-quadruplex

DNAzyme system shown in Figure 1. The kinetic scheme of the reactions associated with the time-dependent concentration changes during the dissipative transitions are summarized in the above equations. Knowing the time-dependent concentration changes of G_1, during the dissipative transitions, we computationally simulated the time-dependent concentration changes by using Matlab R2019b.

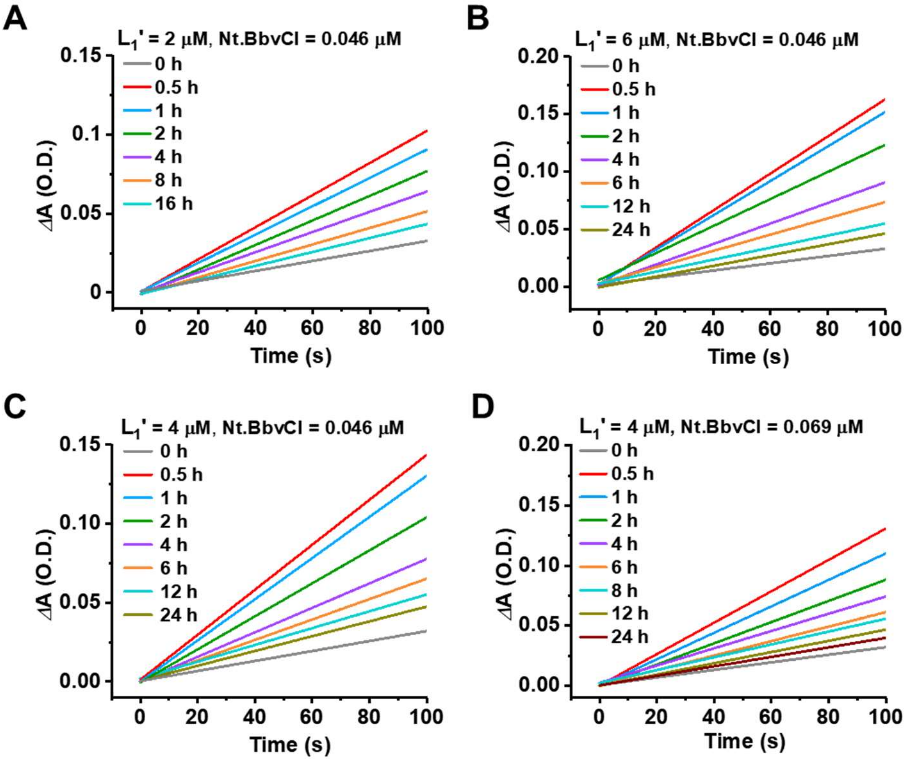

Figure S3. Time-dependent absorbance changes of $ABTS^{\bullet-}$ catalyzed by the transient dissipative hemin/G-quadruplex DNAzyme system at time intervals following the addition of variable concentrations of L_1' and Nt.BbvCI: (A) $L_1' = 2$ μM, Nt.BbvCI = 0.046 μM; (B) $L_1' = 6$ μM, Nt.BbvCI = 0.046 μM; (C) $L_1' = 4$ μM, Nt.BbvCI = 0.046 μM; (D) $L_1' = 4$ μM, Nt.BbvCI = 0.069 μM.

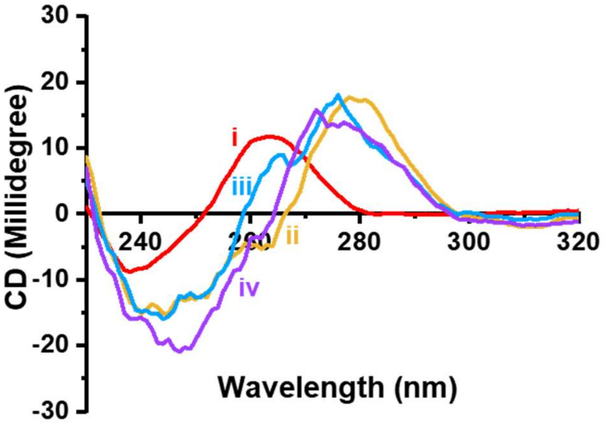

Figure S4. CD spectra corresponding to: (i) the G-quadruplex structure (G_1 = 1.2 μM); (ii) state I (G_1/C_1 = 1.2 μM, L_1/T_1 = 1.2 μM, Nt.BbvCI = 0.058 μM) prior to adding the trigger L_1' (0 h); (iii) the G-quadruplex system (G_1/C_1 = 1.2 μM, L_1/T_1 = 1.2 μM, no Nt.BbvCI) with the addition of L_1' (5 μM); (iv) the transient dissipative G-quadruplex system (G_1/C_1 = 1.2 μM, L_1/T_1 = 1.2 μM, Nt.BbvCI = 0.058 μM) with L_1' (5 μM) after reacting at 33 °C for 12 h. All of the systems were prepared in 1 × rCutSmart buffer including 50 mM of K^+.

As shown in the CD spectrum (i) of Figure S4, the G-quadruplex assembled by G_1 exhibits a dominant positive CD peak at 265 nm and a negative peak at 240 nm, demonstrating the typical signature of a parallel G quadruplex structure in the presence of K^+. For the CD spectrum of (ii) the reaction module in prior to adding the trigger L_1' (i.e., 0 h), no positive peak of G-quadruplex at 265 nm is observed in the presence of K^+ because G_1 is present as the double-stranded G_1/C_1 in the initial system. Upon adding the trigger L_1' into the system without Nt.BbvCI, a distinct positive peak at 265 nm is observed in the CD spectrum (iii), indicating that the addition of L_1' triggers the transformation of G_1 into the parallel G-quadruplex structure. After subjecting the system to both Nt.BbvCI and L_1' for 12 h, the positive peak at 265 nm disappears from the CD spectrum (iv), which demonstrates the dissipative depletion of G-quadruplex and the transformation of G_1 back into the double-stranded structure of G_1/C_1 in the initial state I.

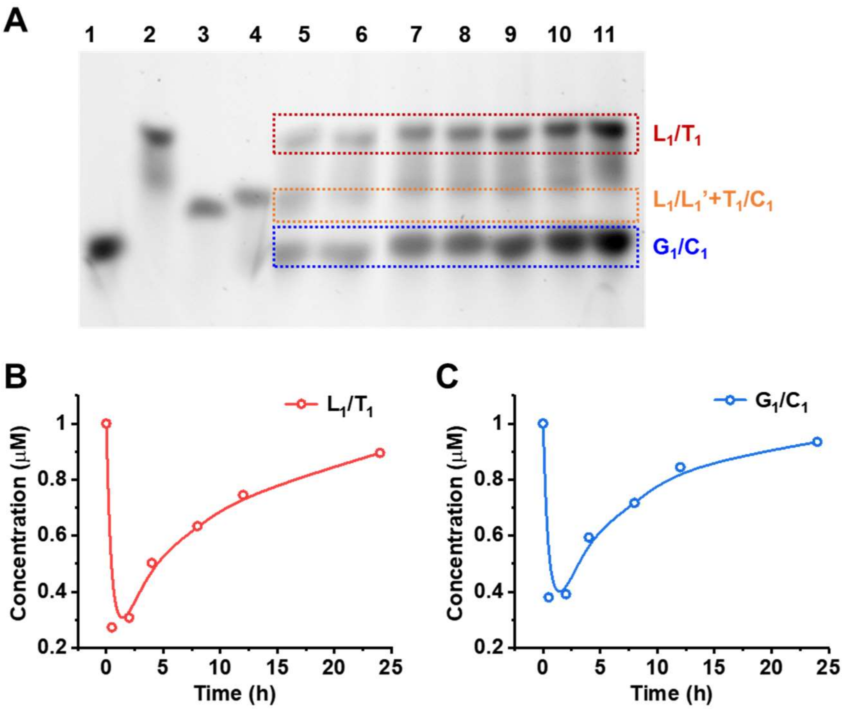

Figure S5. Electrophoretic separation and quantitative analysis of the transient dissipative G-quadruplex system shown in Figure 1 at time intervals following addition of L_1' (4.0 μM) into the system (G_1/C_1 = 1.0 μM, L_1/T_1 = 1.0 μM, Nt.BbvCI = 0.046 μM) at 33 °C. (A) Gel electrophoresis photo graph. Lane 1 – G_1/C_1; Lane 2 – L_1/T_1; Lane 3 – L_1/L_1'; Lane 4 – T_1/C_1; Lanes 5–11 separated bands observed upon treatment of the system to L_1' at different time intervals: Lane 5 – 0.5 h; Lane 6 – 2 h; Lane 7 – 4 h; Lane 8 – 8 h; Lane 9 – 12 h; Lane 10 – 24 h; Lane 11 – 0 h. (B) Time-dependent concentration changes of L_1/T_1. (C) Time-dependent concentration changes of G_1/C_1. The concentrations were quantitatively evaluated by using the Image J software to compare the intensity of the band to that of the corresponding bands, L_1/T_1 and G_1/C_1, at 0 h with a known concentration of 1.0 μM.

As shown in Figure S5A, at 0 h (lane 11), no bands of L_1/L_1' or T_1/C_1 were observed. Upon subjecting the reaction module, state I, to the trigger L_1' for 0.5 h (lane 5), the bands of L_1/L_1' and T_1/C_1 were observed, and the band intensities of L_1/T_1 and G_1/C_1 significantly decreased in comparison with that of L_1/T_1 and G_1/C_1 at 0 h (lane 11). This result demonstrates that the displacement of L_1/T_1 by the fuel strand L_1' yields L_1/L_1' and releases T_1 to displace C_1 from G_1/C_1. During the dissipative process from 2 h to 24 h, the band intensities of L_1/L_1' and T_1/C_1 gradually decreased and eventually disappeared due to the cleavage of L_1' in L_1/L_1' by Nt.BbvCI nicking enzyme and the displacement of T_1/C_1 by the released L_1. At the same time, the band intensities of L_1/T_1 and G_1/C_1 gradually increased and eventually reached the intensities comparable to that

of their initial bands. These results indicate that the system undergoes a process of transient dissipative transformation of the DNA structures.

Since the bands of L_1/L_1' and T_1/C_1 overlapped, neither could be quantitatively evaluated. Therefore, we evaluated the time-dependent concentration changes of L_1/T_1 and G_1/C_1 by comparing their band intensities at time intervals to their band intensities at 0 h (1.0 μM) using Image J software. As summarized in Figure S5B, the concentration of L_1/T_1 decreased to 0.27 μM upon subjecting the system to the fuel strand L_1' for 0.5 h. During the dissipative process from 2 h to 24 h, the concentration of L_1/T_1 gradually increased and finally returned to a concentration of 0.90 μM. Similar results were obtained for G_1/C_1 (Figure S5C). Upon mixing the system with L_1' for 0.5 h, the concentration of G_1/C_1 decreased to 0.38 μM. After that, the concentration of G_1/C_1 gradually increased and finally reached a concentration of 0.93 μM. These results indicate the system exhibits a transient dissipative behavior to transiently form the DNA structures and subsequently return to the original state I.

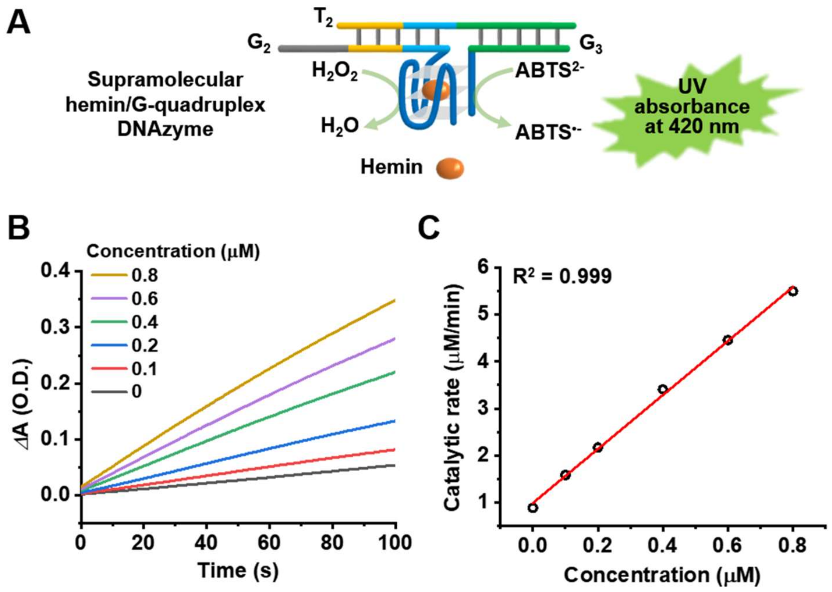

Figure S6. (A) Schematic illustration of the catalysis of $ABTS^{2-}$ to $ABTS^{\bullet-}$ by the supramolecular T_2/G_2+G_3 hemin/G-quadruplex DNAzyme in the presence of H_2O_2. (B) The time-dependent absorbance changes of $ABTS^{\bullet-}$ upon the addition of variable concentrations of T_2 to bind G_2 and G_3. (C) The derived calibration curve corresponding to the catalytic rate of $ABTS^{\bullet-}$ at different standard concentrations of T_2/G_2+G_3 hemin/G-quadruplex DNAzyme. $ABTS^{2-}$ concentration was 0.25 mM and H_2O_2 concentration was 0.50 mM.

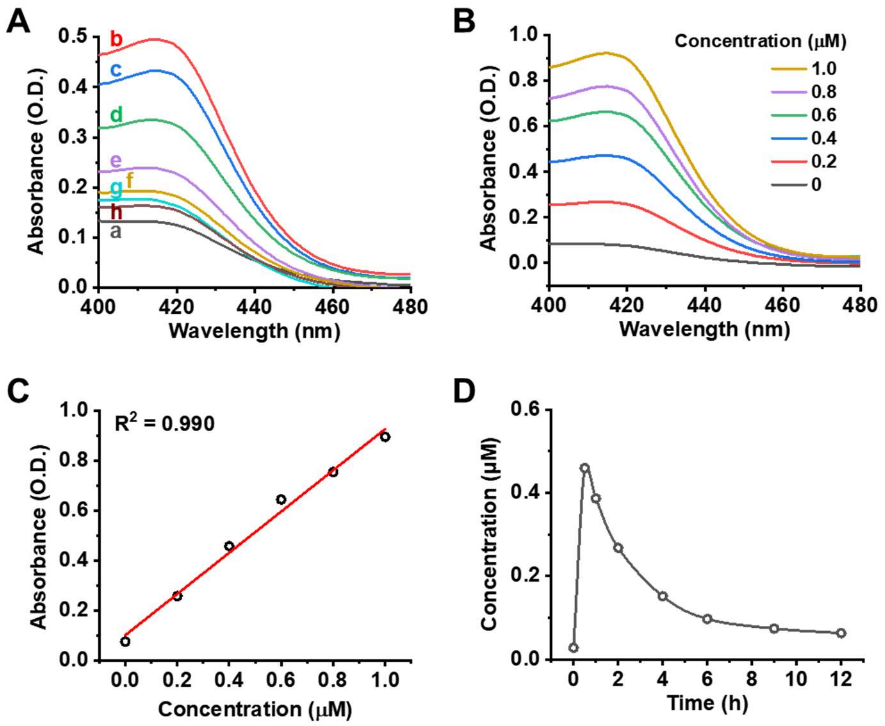

Figure S7. (A) Absorbance spectra of $ABTS^{\bullet-}$ generated by the hemin/G-quadruplex T_2/G_2+G_3 DNAzyme formed at different time intervals following the activation of the transient reaction module: (a) 0 h, (b) 0.5 h, (c) 1 h, (d) 2 h, (e) 4 h, (f) 6 h, (g) 9 h, (h) 12 h. The absorption spectra were obtained at 100 s after mixing aliquots of the reaction solution with $ABTS^{2-}$ and H_2O_2. (B) Absorption spectra of $ABTS^{\bullet-}$ mixed with variable known concentrations of T_2/G_2+G_3 hemin/G-quadruplex DNAzyme in the presence of H_2O_2 for 100 s. (C) The derived calibration curve corresponding to the absorbance of $ABTS^{\bullet-}$ at 420 nm at different standard concentrations of T_2/G_2+G_3 hemin/G-quadruplex DNAzyme. (D) The dotted curve shows the transient concentration of the hemin/G-quadruplex T_2/G_2+G_3 DNAzyme. Experimental results correspond to the reaction module consisting of: L_2/T_2 = 1 μM, G_2 = 1 μM, G_3 = 1 μM, hemin = 1 μM, Nt.BbvCI = 0.069 μM, L_1' = 4 μM in 1 × CutSmart buffer. The concentrations of $ABTS^{2-}$ and H_2O_2 in final system was 0.25 mM and 0.50 mM.

Kinetic equations of the transient dissipative hemin/G-quadruplex DNAzyme system shown in Figure 2:

(8) $L_2 + T_2 \underset{k_{-8}}{\overset{k_8}{\rightleftarrows}} L_2T_2$

(9) $L_2T_2 + L_2' \underset{k_{-9}}{\overset{k_9}{\rightleftarrows}} L_2L_2' + T_2$

(10) $T_2 + G_2 + G_3 \underset{k_{-10}}{\overset{k_{10}}{\rightleftarrows}} T_2G_2G_3$

(11) $L_2L_2' + E \underset{k_{-11}}{\overset{k_{11}}{\rightleftarrows}} L_2L_2'E$

(12) $L_2L_2'E \xrightarrow{k_{12}} E + L_2L_{2-1}'L_{2-2}'$

(13) $L_2L_{2-1}'L_{2-2}' \underset{k_{-13}}{\overset{k_{13}}{\rightleftarrows}} L_2 + L_{2-1}' + L_{2-2}'$

Derivatives:

$$\frac{dL_2}{dt} = k_{-8}[L_2T_2] - k_8[L_2][T_2] + k_{13}[L_2L_{2-1}'L_{2-2}'] - k_{-13}[L_2][L_{2-1}'][L_{2-2}']$$

$$\frac{dT_2}{dt} = k_{-8}[L_2T_2] - k_8[L_2][T_2] + k_9[L_2T_2][L_2'] - k_{-9}[L_2L_2'][T_2] + k_{-10}[T_2G_2G_3] - k_{10}[T_2][G_2][G_3]$$

$$\frac{dL_2T_2}{dt} = k_8[L_2][T_2] - k_{-8}[L_2T_2] + k_{-9}[L_2L_2'][T_2] - k_9[L_2T_2][L_2']$$

$$\frac{dL_2'}{dt} = k_{-9}[L_2L_2'][T_2] - k_9[L_2T_2][L_2']$$

$$\frac{dL_2L_2'}{dt} = k_9[L_2T_2][L_2'] - k_{-9}[L_2L_2'][T_2] + k_{-11}[L_2L_2'E] - k_{11}[L_2L_2'][E]$$

$$\frac{dG_2}{dt} = k_{-10}[T_2G_2G_3] - k_{10}[T_2][G_2][G_3]$$

$$\frac{dG_3}{dt} = k_{-10}[T_2G_2G_3] - k_{10}[T_2][G_2][G_3]$$

$$\frac{dT_2G_2G_3}{dt} = k_{10}[T_2][G_2][G_3] - k_{-10}[T_2G_2G_3]$$

$$\frac{dE}{dt} = k_{-11}[L_2L_2'E] - k_{11}[L_2L_2'][E] + k_{12}[L_2L_2'E]$$

$$\frac{dL_2L_2'E}{dt} = k_{11}[L_2L_2'][E] - k_{-11}[L_2L_2'E] - k_{12}[L_2L_2'E]$$

$$\frac{dL_2L_{2-1}'L_{2-2}'}{dt} = k_{12}[L_2L_2'E] - k_{13}[L_2L_{2-1}'L_{2-2}'] + k_{-13}[L_2][L_{2-1}'][L_{2-2}']$$

$$\frac{dL_{2-1}'}{dt} = k_{13}[L_2L_{2-1}'L_{2-2}'] - k_{-13}[L_2][L_{2-1}'][L_{2-2}']$$

$$\frac{dL_{2-2}'}{dt} = k_{13}[L_2L_{2-1}'L_{2-2}'] - k_{-13}[L_2][L_{2-1}'][L_{2-2}']$$

Figure S8. Computational simulation of the dissipative system shown in Figure 2. The kinetic scheme of the reactions associated with the time-dependent concentration changes during the dissipative transitions is summarized in the above equations. Knowing the time-dependent concentration changes of T_2/G_2+G_3, during the dissipative transitions, we computationally simulated the time-dependent concentration changes by using Matlab R2019b.

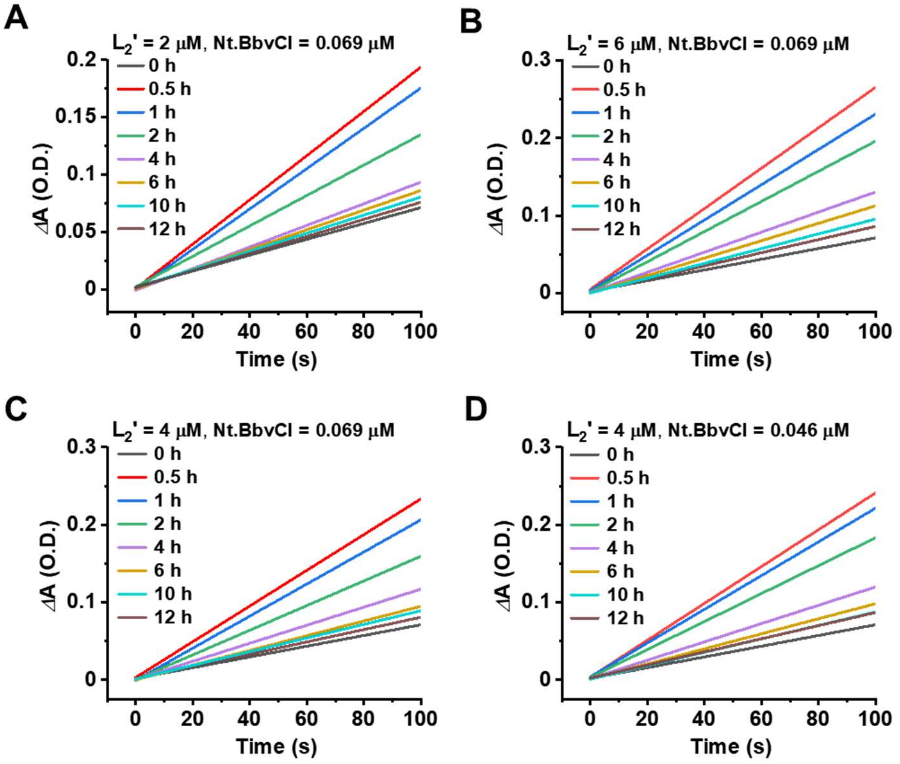

Figure S9. Time-dependent absorbance changes of $ABTS^{\bullet-}$ catalyzed by the transient dissipative hemin/G-quadruplex DNAzyme shown in Figure 2 at time intervals following addition of variable concentrations of the trigger L_2' and Nt.BbvCI: (A) L_2' = 2 μM, Nt.BbvCI = 0.069 μM; (B) L_2' = 6 μM, Nt.BbvCI = 0.069 μM; (C) L_2' = 4 μM, Nt.BbvCI = 0.069 μM; (D) L_2' = 4 μM, Nt.BbvCI = 0.046 μM.

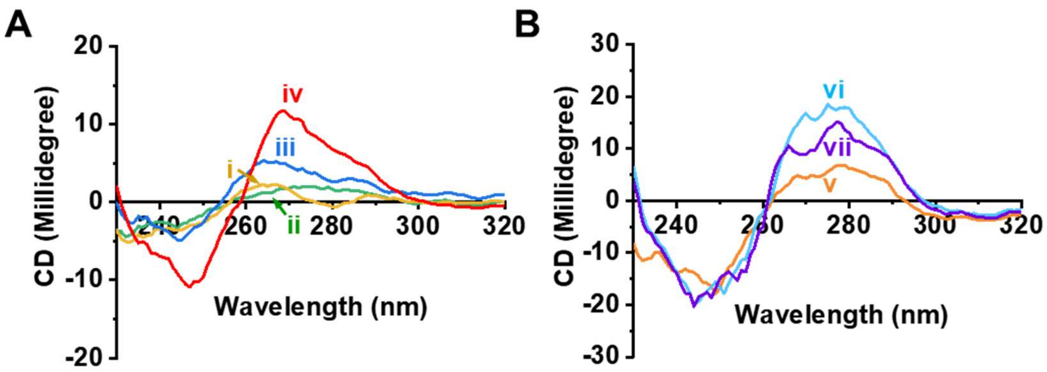

Figure S10. (A) CD spectra corresponding to: (i) G_2 (1 μM); (ii) G_3 (1 μM); (iii) G_2+G_3 (1 μM); (iv) T_2/G_2+G_3 (1 μM). (B) CD spectra corresponding to: (v) the state II (G_2 = 1.2 μM, G_3 = 1.2 μM, L_2/T_2 = 1.2 μM, Nt.BbvCI = 0.058 μM) prior to adding the trigger L_2' (0 h); (vi) the supramolecular G-quadruplex system (G_2 = 1.2 μM, G_3 = 1.2 μM, L_2/T_2 = 1.2 μM, no Nt.BbvCI) with the fuel strand L_2' (5 μM); (vii) the transient dissipative G-quadruplex system (G_2 = 1.2 μM, G_3 = 1.2 μM, L_2/T_2 = 1.2 μM, Nt.BbvCI = 0.058 μM) with L_2' (5 μM) after reacting at 33 °C for 4 h. All of the systems were prepared in 1 × rCutSmart buffer including 50 mM of K^+.

Figure S10(A) shows the CD spectra corresponding to (i) G_2, (ii) G_3, (iii) G_2+G_3, and (iv) T_2/G_2+G_3, respectively. T_2/G_2+G_3 exhibits strong positive and negative CD peaks indicating the distinctive secondary structure of the supramolecular T_2/G_2+G_3 complex, whilst the spectra of the two G subunits in the absence of T_2 result in only very weak CD signals. In the CD spectrum (v) of Figure S10(B) corresponding to the state II prior to adding trigger L_2' (i.e., 0 h), very weak CD intensity is observed, because the ability of G_2 and G_3 to assemble in the absence of free T_2 is low (as demonstrated in the control spectrum, iii, of the two subunits). As shown in (vi), upon adding L_2' into the system without Nt.BbvCI, the peak intensity increases significantly, demonstrating that the addition of L_2' successfully triggers the formation of the supramolecular T_2/G_2+G_3 complex. After subjecting the system to both Nt.BbvCI and L_2' for 4 h, the peak intensities clearly decrease (vii), demonstrating that the system exhibits dissipative behavior, with the concentration of T_2/G_2+G_3 depleting over time due to the cleavage of L_2' in L_2/L_2' by the Nt.BbvCI nicking enzyme, resulting in the displacement of T_2/G_2+G_3 by the released L_2.

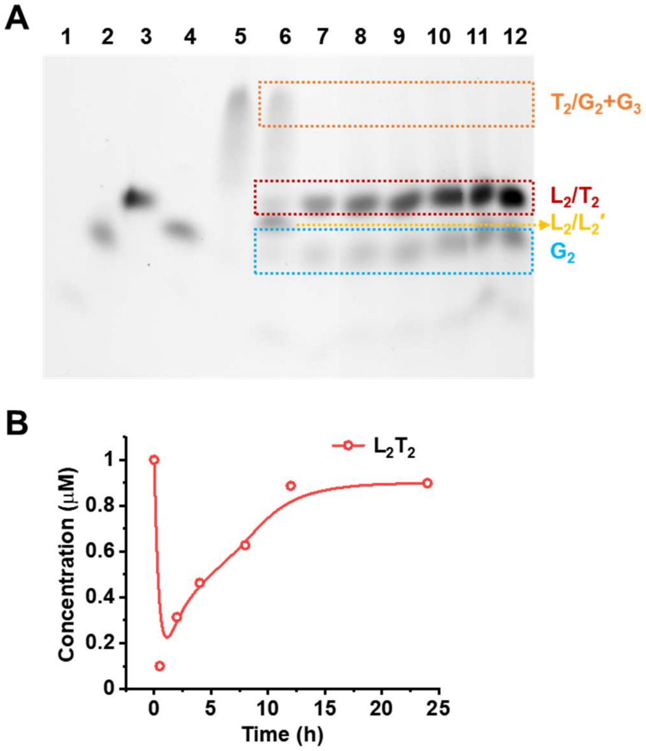

Figure S11. Electrophoretic separation and quantitative analysis of the transient dissipative G-quadruplex system shown in Figure 2 at time intervals following mixing the system (L_2/T_2 = 1 μM, G_2 = 1 μM, G_3 = 1 μM, Nt.BbvCI = 0.046 μM) with the trigger L_2' (4.0 μM) at 33 °C. (A) Gel electrophoresis photo graph. Lane 1 – G_3; Lane 2 – G_2; Lane 3 – L_2/T_2; Lane 4 – L_2/L_2'; Lane 5 – T_2/G_2+G_3; Lanes 6–12 separated bands observed upon treatment of the system to the fuel strand L_2' for different time intervals: Lane 6 – 0.5 h; Lane 7 – 2 h; Lane 8 – 4 h; Lane 9 – 8 h; Lane 10 – 12 h; Lane 11 – 24 h; Lane 12 – 0 h. (B) Time-dependent concentration changes of L_2/T_2. The concentration was quantitatively evaluated by using Image J software to compare the intensity of L_2/T_2 band to that of the corresponding band at 0 h with a known concentration of 1.0 μM.

As shown in Figure S11A, at 0 h (lane 12), no bands of L_2/L_2' or T_1/G_2+G_3 were observed. Upon subjecting the reaction module, state II, to the trigger L_2' for 0.5 h (lane 6), the bands of L_2/L_2' and T_2/G_2+G_3 were observed, and the band intensities of L_2/T_2 and G_2 with a hairpin structure significantly decreased in comparison with that of L_2/T_2 and G_2 at 0 h (lane 12). This result demonstrates that the displacement of L_2/T_2 by the fuel strand L_2' yields L_2/L_2' and releases T_2 to combine G_2 and G_3 together. During the dissipative process from 2 h to 24 h, the band intensities of L_2/L_2' and T_2/G_2+G_3 decreased and eventually disappeared due to the cleavage of L_2' in L_2/L_2' by Nt.BbvCI

nicking enzyme and the displacement of T_2/G_2+G_3 by the released L_2. At the same time, the band intensities of L_2/T_2 and G_2 gradually increased and eventually reached intensities comparable to that of their initial bands. These results indicate that the system undergoes a process of transient dissipative transformation of DNA structures.

Since the bands of L_2/T_2 can be clearly observed for all the time intervals, we evaluated the time-dependent concentration changes of L_2/T_2 by comparing their band intensities at time intervals to their band intensities at 0 h (1.0 μM) using Image J software. As summarized in Figure S11B, the concentration of L_2/T_2 decreased to 0.10 μM upon subjecting the system to the fuel strand L_2' for 0.5 h. During the dissipative process from 2 h to 24 h, the concentration of L_2/T_2 gradually increased and finally returned to a concentration of 0.90 μM. These results indicate the system shown in Figure 2 has a transient and dissipative behavior to transform DNA structures and the ability to regenerate the original system.

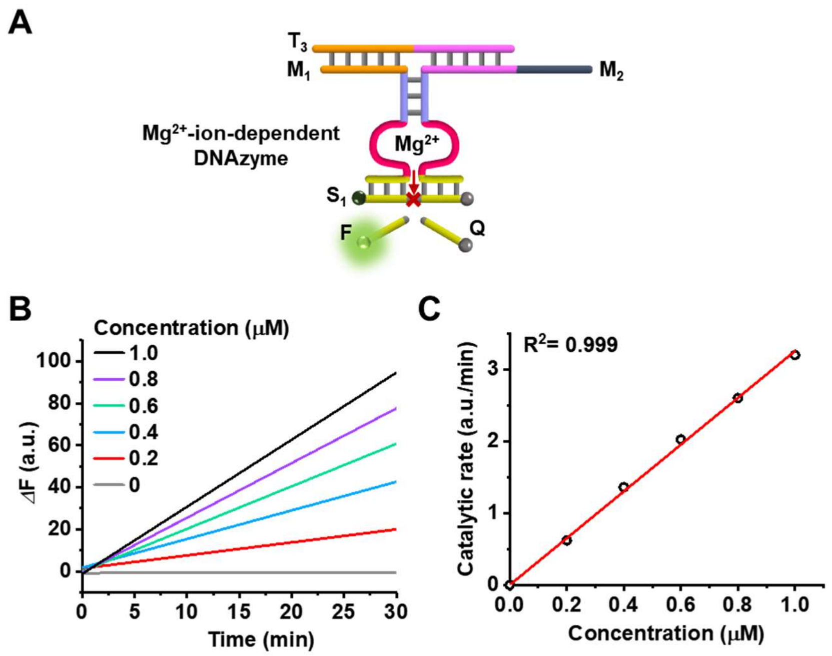

Figure S12. (A) Schematic illustration of the cleavage of the fluorophore/quencher-modified substrate (S_1) by the Mg^{2+}-ion-dependent DNAzyme. (B) Time-dependent fluorescence changes generated from the cleavage of S_1 by the Mg^{2+}-ion-dependent DNAzyme upon the addition of variable concentrations of T_3 to bind M_1 and M_2. (C) The derived calibration curve corresponding to the catalytic rate at different standard concentrations of T_3/M_1+M_2 Mg^{2+}-ion-dependent DNAzyme.

Kinetic equations of the transient Mg^{2+}-ion-dependent DNAzyme shown in Figure 3:

(14) $L_3 + T_3 \underset{k_{-14}}{\overset{k_{14}}{\rightleftarrows}} L_3T_3$

(15) $L_3T_3 + L_3' \underset{k_{-15}}{\overset{k_{15}}{\rightleftarrows}} L_3L_3' + T_3$

(16) $T_3 + M_1 + M_2 \underset{k_{-16}}{\overset{k_{16}}{\rightleftarrows}} T_3M_1M_2$

(17) $L_3L_3' + E \underset{k_{-17}}{\overset{k_{17}}{\rightleftarrows}} L_3L_3'E$

(18) $L_3L_3'E \xrightarrow{k_{18}} E + L_3L_{3-1}'L_{3-2}'$

(19) $L_3L_{3-1}'L_{3-2}' \underset{k_{-19}}{\overset{k_{19}}{\rightleftarrows}} L_3 + L_{3-1}' + L_{3-2}'$

Derivatives:

$$\frac{dL_3}{dt} = k_{-14}[L_3T_3] - k_{14}[L_3][T_3] + k_{19}[L_3L_{3-1}'L_{3-2}'] - k_{-19}[L_3][L_{3-1}'][L_{3-2}']$$

$$\frac{dT_3}{dt} = k_{-14}[L_3T_3] - k_{14}[L_3][T_3] + k_{15}[L_3T_3][L_3'] - k_{-15}[L_3L_3'][T_3] + k_{-16}[T_3M_1M_2] - k_{16}[T_3][M_1][M_2]$$

$$\frac{dL_3T_3}{dt} = k_{14}[L_3][T_3] - k_{-14}[L_3T_3] + k_{-15}[L_3L_3'][T_3] - k_{15}[L_3T_3][L_3']$$

$$\frac{dL_3'}{dt} = k_{-15}[L_3L_3'][T_3] - k_{15}[L_3T_3][L_3']$$

$$\frac{dL_3L_3'}{dt} = k_{15}[L_3T_3][L_3'] - k_{-15}[L_3L_3'][T_3] + k_{-17}[L_3L_3'E] - k_{17}[L_3L_3'][E]$$

$$\frac{dM_1}{dt} = k_{-16}[T_3M_1M_2] - k_{16}[T_3][M_1][M_2]$$

$$\frac{dM_2}{dt} = k_{-16}[T_3M_1M_2] - k_{16}[T_3][M_1][M_2]$$

$$\frac{dT_3M_1M_2}{dt} = k_{16}[T_3][M_1][M_2] - k_{-16}[T_3M_1M_2]$$

$$\frac{dE}{dt} = k_{-17}[L_3L_3'E] - k_{17}[L_3L_3'][E] + k_{18}[L_3L_3'E]$$

$$\frac{dL_3L_3'E}{dt} = k_{17}[L_3L_3'][E] - k_{-17}[L_3L_3'E] - k_{18}[L_3L_3'E]$$

$$\frac{dL_3L_{3-1}'L_{3-2}'}{dt} = k_{18}[L_3L_3'E] - k_{19}[L_3L_{3-1}'L_{3-2}'] + k_{-19}[L_3][L_{3-1}'][L_{3-2}']$$

$$\frac{dL_{3-1}'}{dt} = k_{19}[L_3L_{3-1}'L_{3-2}'] - k_{-19}[L_3][L_{3-1}'][L_{3-2}']$$

$$\frac{dL_{3-2}'}{dt} = k_{19}[L_3L_{3-1}'L_{3-2}'] - k_{-19}[L_3][L_{3-1}'][L_{3-2}']$$

Figure S13. Computational simulation of the transient Mg^{2+}-ion-dependent DNAzyme shown in Figure 3. The kinetic scheme of the reactions associated with the time-dependent concentration changes during the dissipative transitions is summarized in the above equations. Knowing the time-dependent concentration changes of T_3/M_1+M_2, during the dissipative transitions, we computationally simulated the time-dependent concentration changes by using Matlab R2019b.

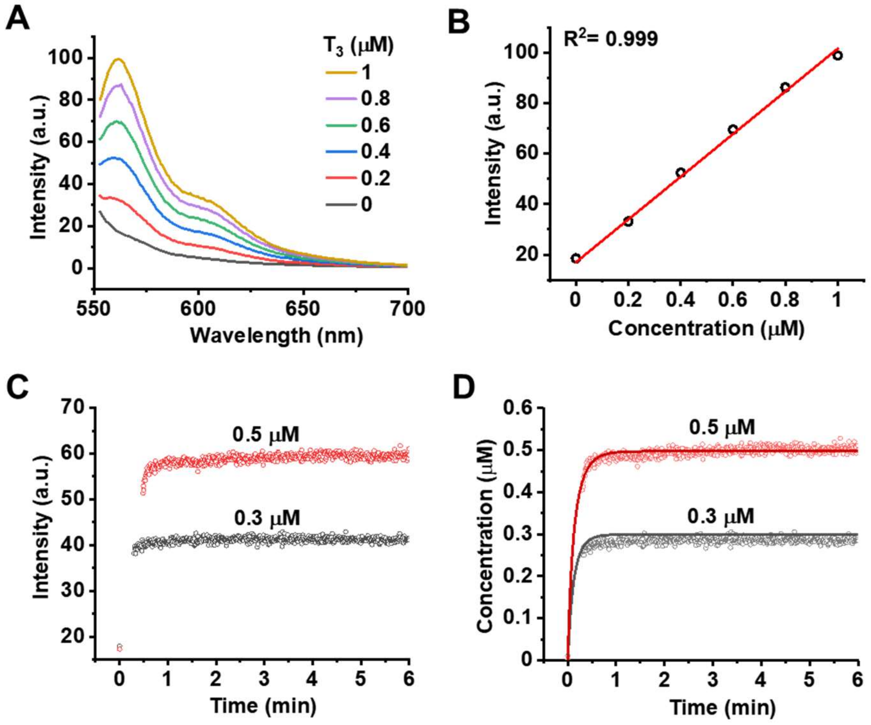

Figure S14. (A) The fluorescence spectra of Cy3-labeled L_3/BHQ2-labeled L_3' (1 μM) upon the addition of variable concentrations of T_3. (B) The derived calibration curve corresponding to the fluorescence intensity of the Cy3 (λ_{em} = 560 nm) at different concentrations of T_3. (C) Time-dependent fluorescence changes upon subjecting the labeled L_3/ L_3' to the different concentrations of T_3: 0.3 μM (black) and 0.5 μM (red). (D) Time-dependent concentration changes of T_3/L_3 upon subjecting the labeled L_3/L_3' to the different concentrations of T_3: 0.3 μM (black) and 0.5 μM (red). Dots correspond to the experimental data derived from the results shown in (C) by using the calibration curve (B). Solid curves correspond to the computationally simulated kinetic profiles.

Experimental evaluation of the rate constants k_{15} and k_{-15} of the kinetic scheme in Figures S13 and S17–S19

To evaluate the rate constants k_{15} and k_{-15} appearing in the kinetic scheme (equation 15 in Figures S13 and S17–S19), we subjected the L_3/L_3' duplex (1.0 μM) formed by the Cy3-labeled L_3 and BHQ2-labeled L_3' to two concentrations of T_3 (0.3 μM and 0.5 μM). The time-dependent concentration changes of T_3/L_3 were evaluated by following the time-dependent fluorescence changes of Cy3 at λ_{em} = 560 nm and translating them into concentrations by applying an appropriate calibration curve, Figure S14. From the kinetic profiles and using the Matlab R2019b program, the respective k_{15} = 0.06 μM^{-1} min^{-1} and k_{-15} = 9.0 μM^{-1} min^{-1} were derived.

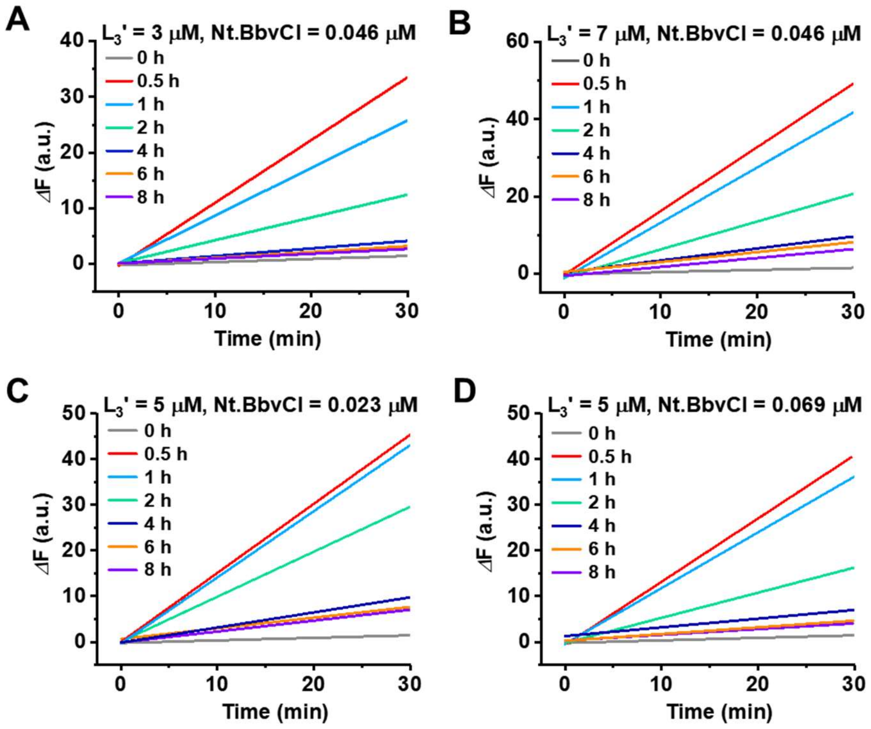

Figure S15. Time-dependent fluorescence changes generated from the cleavage of S_1 by the transiently-formed Mg^{2+}-ion-dependent DNAzyme at time intervals following addition of variable concentrations of L_3' and Nt.BbvCI: (A) L_3' = 3 μM, Nt.BbvCI = 0.046 μM; (B) L_3' = 7 μM, Nt.BbvCI = 0.046 μM; (C) L_3' = 5 μM, Nt.BbvCI = 0.023 μM; (D) L_3' = 5 μM, Nt.BbvCI = 0.069 μM.

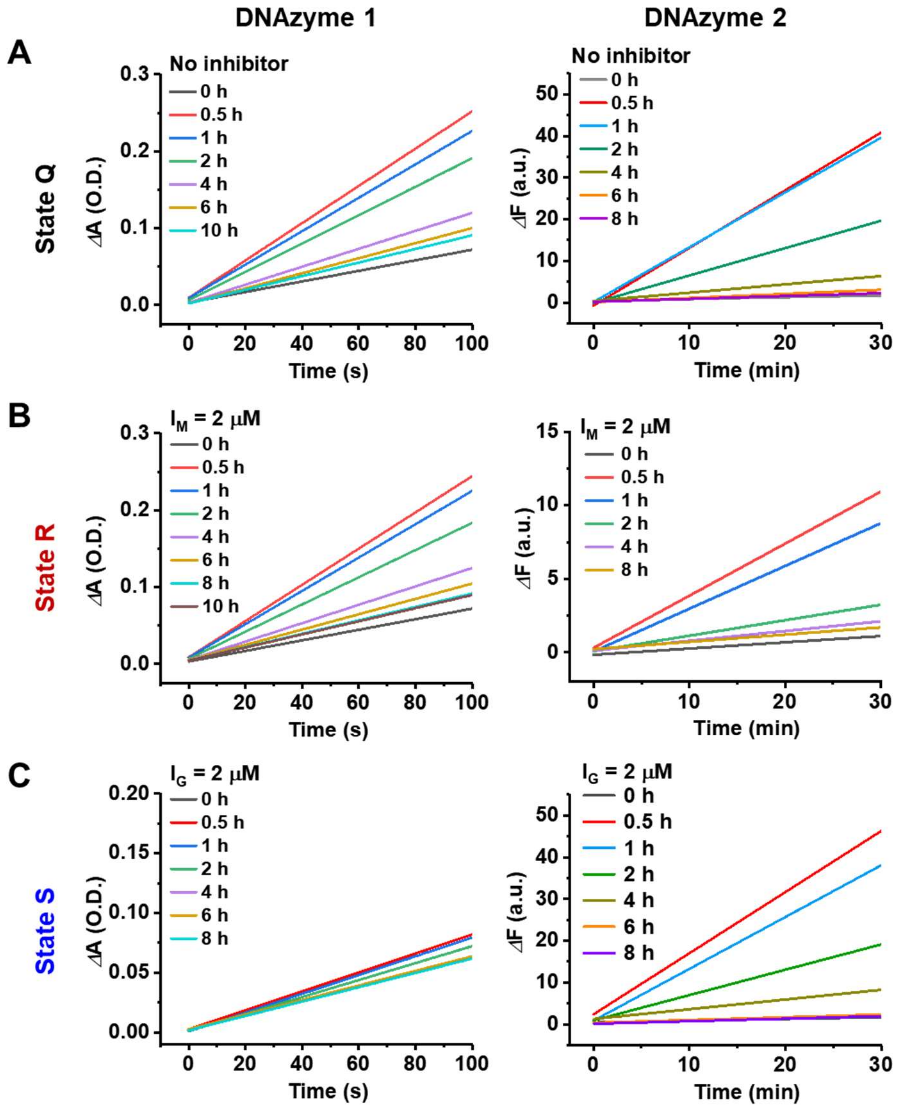

Figure S16. Time-dependent absorbance changes of $ABTS^{\bullet -}$ and time-dependent fluorescence changes generated from the cleavage of S_1 catalyzed by the gated transient DNAzymes: (A) State Q, inhibitors $I_G = 0$ μM, $I_M = 0$ μM; (B) State R, inhibitors $I_G = 0$ μM, $I_M = 2$ μM; (C) State S, inhibitors $I_G = 2$ μM, $I_M = 0$ μM. For all the gated DNAzyme system: $L_2/T_2 = 1$ μM, $L_3/T_3 = 1$ μM, $G_2 = 1$ μM, $G_3 = 1$ μM, $M_1 = 1$ μM, $M_2 = 1$ μM, hemin = 1 μM, Nt.BbvCI = 0.069 μM, $L_2' = 4$ μM, $L_3' = 5$ μM, T = 33 °C.

Kinetic equations of the parallel, non-gated DNAzymes (state Q) shown in Figure 4:

(8) $L_2 + T_2 \underset{k_{-8}}{\overset{k_8}{\rightleftarrows}} L_2T_2$

(9) $L_2T_2 + L_2' \underset{k_{-9}}{\overset{k_9}{\rightleftarrows}} L_2L_2' + T_2$

(10) $T_2 + G_2 + G_3 \underset{k_{-10}}{\overset{k_{10}}{\rightleftarrows}} T_2G_2G_3$

(11) $L_2L_2' + E \underset{k_{-11}}{\overset{k_{11}}{\rightleftarrows}} L_2L_2'E$

(12) $L_2L_2'E \xrightarrow{k_{12}} E + L_2L_{2-1}'L_{2-2}'$

(13) $L_2L_{2-1}'L_{2-2}' \underset{k_{-13}}{\overset{k_{13}}{\rightleftarrows}} L_2 + L_{2-1}' + L_{2-2}'$

(14) $L_3 + T_3 \underset{k_{-14}}{\overset{k_{14}}{\rightleftarrows}} L_3T_3$

(15) $L_3T_3 + L_3' \underset{k_{-15}}{\overset{k_{15}}{\rightleftarrows}} L_3L_3' + T_3$

(16) $T_3 + M_1 + M_2 \underset{k_{-16}}{\overset{k_{16}}{\rightleftarrows}} T_3M_1M_2$

(17) $L_3L_3' + E \underset{k_{-17}}{\overset{k_{17}}{\rightleftarrows}} L_3L_3'E$

(18) $L_3L_3'E \xrightarrow{k_{18}} E + L_3L_{3-1}'L_{3-2}'$

(19) $L_3L_{3-1}'L_{3-2}' \underset{k_{-19}}{\overset{k_{19}}{\rightleftarrows}} L_3 + L_{3-1}' + L_{3-2}'$

Derivatives:

$$\frac{dL_2}{dt} = k_{-8}[L_2T_2] - k_8[L_2][T_2] + k_{13}[L_2L_{2-1}'L_{2-2}'] - k_{-13}[L_2][L_{2-1}'][L_{2-2}']$$

$$\frac{dT_2}{dt} = k_{-8}[L_2T_2] - k_8[L_2][T_2] + k_9[L_2T_2][L_2'] - k_{-9}[L_2L_2'][T_2] + k_{-10}[T_2G_2G_3] - k_{10}[T_2][G_2][G_3]$$

$$\frac{dL_2T_2}{dt} = k_8[L_2][T_2] - k_{-8}[L_2T_2] + k_{-9}[L_2L_2'][T_2] - k_9[L_2T_2][L_2']$$

$$\frac{dL_2'}{dt} = k_{-9}[L_2L_2'][T_2] - k_9[L_2T_2][L_2']$$

$$\frac{dL_2L_2'}{dt} = k_9[L_2T_2][L_2'] - k_{-9}[L_2L_2'][T_2] + k_{-11}[L_2L_2'E] - k_{11}[L_2L_2'][E]$$

$$\frac{dG_2}{dt} = k_{-10}[T_2G_2G_3] - k_{10}[T_2][G_2][G_3]$$

$$\frac{dG_3}{dt} = k_{-10}[T_2G_2G_3] - k_{10}[T_2][G_2][G_3]$$

$$\frac{dT_2G_2G_3}{dt} = k_{10}[T_2][G_2][G_3] - k_{-10}[T_2G_2G_3]$$

$$\frac{dE}{dt} = k_{-11}[L_2L_2'E] - k_{11}[L_2L_2'][E] + k_{12}[L_2L_2'E] + k_{-17}[L_3L_3'E] - k_{17}[L_3L_3'][E] + k_{18}[L_3L_3'E]$$

$$\frac{dL_2L_2'E}{dt} = k_{11}[L_2L_2'][E] - k_{-11}[L_2L_2'E] - k_{12}[L_2L_2'E]$$

$$\frac{dL_2L_{2-1}'L_{2-2}'}{dt} = k_{12}[L_2L_2'E] - k_{13}[L_2L_{2-1}'L_{2-2}'] + k_{-13}[L_2][L_{2-1}'][L_{2-2}']$$

$$\frac{dL_{2-1}'}{dt} = k_{13}[L_2L_{2-1}'L_{2-2}'] - k_{-13}[L_2][L_{2-1}'][L_{2-2}']$$

$$\frac{dL_{2-2}'}{dt} = k_{13}[L_2L_{2-1}'L_{2-2}'] - k_{-13}[L_2][L_{2-1}'][L_{2-2}']$$

$$\frac{dL_3}{dt} = k_{-14}[L_3T_3] - k_{14}[L_3][T_3] + k_{19}[L_3L_{3-1}'L_{3-2}'] - k_{-19}[L_3][L_{3-1}'][L_{3-2}']$$

$$\frac{dT_3}{dt} = k_{-14}[L_3T_3] - k_{14}[L_3][T_3] + k_{15}[L_3T_3][L_3'] - k_{-15}[L_3L_3'][T_3] + k_{-16}[T_3M_1M_2] - k_{16}[T_3][M_1][M_2]$$

$$\frac{dL_3T_3}{dt} = k_{14}[L_3][T_3] - k_{-14}[L_3T_3] + k_{-15}[L_3L_3'][T_3] - k_{15}[L_3T_3][L_3']$$

$$\frac{dL_3'}{dt} = k_{-15}[L_3L_3'][T_3] - k_{15}[L_3T_3][L_3']$$

$$\frac{dL_3L_3'}{dt} = k_{15}[L_3T_3][L_3'] - k_{-15}[L_3L_3'][T_3] + k_{-17}[L_3L_3'E] - k_{17}[L_3L_3'][E]$$

$$\frac{dM_1}{dt} = k_{-16}[T_3M_1M_2] - k_{16}[T_3][M_1][M_2]$$

$$\frac{dM_2}{dt} = k_{-16}[T_3M_1M_2] - k_{16}[T_3][M_1][M_2]$$

$$\frac{dT_3M_1M_2}{dt} = k_{16}[T_3][M_1][M_2] - k_{-16}[T_3M_1M_2]$$

$$\frac{dL_3L_3'E}{dt} = k_{17}[L_3L_3'][E] - k_{-17}[L_3L_3'E] - k_{18}[L_3L_3'E]$$

$$\frac{dL_3L_{3-1}'L_{3-2}'}{dt} = k_{18}[L_3L_3'E] - k_{19}[L_3L_{3-1}'L_{3-2}'] + k_{-19}[L_3][L_{3-1}'][L_{3-2}']$$

$$\frac{dL_{3-1}'}{dt} = k_{19}[L_3L_{3-1}'L_{3-2}'] - k_{-19}[L_3][L_{3-1}'][L_{3-2}']$$

$$\frac{dL_{3-2}'}{dt} = k_{19}[L_3L_{3-1}'L_{3-2}'] - k_{-19}[L_3][L_{3-1}'][L_{3-2}']$$

Figure S17. Computational simulation of the parallel, non-gated DNAzymes (state Q) shown in Figure 4. The kinetic scheme of the reactions associated with the time-dependent concentration changes during the dissipative transitions is summarized in the above equations. Knowing the time-dependent concentration changes of the complexes T_2/G_2+G_3 and T_3/M_1+M_2 during the dissipative transitions, we computationally simulated the time-dependent concentration changes by using Matlab R2019b.

Kinetic equations of the inhibitor I_M-gated transient DNAzymes (state R) shown in Figure 4:

(8) $L_2 + T_2 \underset{k_{-8}}{\overset{k_8}{\rightleftarrows}} L_2T_2$

(9) $L_2T_2 + L_2' \underset{k_{-9}}{\overset{k_9}{\rightleftarrows}} L_2L_2' + T_2$

(10) $T_2 + G_2 + G_3 \underset{k_{-10}}{\overset{k_{10}}{\rightleftarrows}} T_2G_2G_3$

(11) $L_2L_2' + E \underset{k_{-11}}{\overset{k_{11}}{\rightleftarrows}} L_2L_2'E$

(12) $L_2L_2'E \xrightarrow{k_{12}} E + L_2L_{2-1}'L_{2-2}'$

(13) $L_2L_{2-1}'L_{2-2}' \underset{k_{-13}}{\overset{k_{13}}{\rightleftarrows}} L_2 + L_{2-1}' + L_{2-2}'$

(14) $L_3 + T_3 \underset{k_{-14}}{\overset{k_{14}}{\rightleftarrows}} L_3T_3$

(20) $M_2 + I_M \underset{k_{-20}}{\overset{k_{20}}{\rightleftarrows}} M_2I_M$

(15) $L_3T_3 + L_3' \underset{k_{-15}}{\overset{k_{15}}{\rightleftarrows}} L_3L_3' + T_3$

(21) $T_3 + M_2I_M + M_1 \underset{k_{-21}}{\overset{k_{21}}{\rightleftarrows}} T_3M_1M_2 + I_M$

(17) $L_3L_3' + E \underset{k_{-17}}{\overset{k_{17}}{\rightleftarrows}} L_3L_3'E$

(18) $L_3L_3'E \xrightarrow{k_{18}} E + L_3L_{3-1}'L_{3-2}'$

(19) $L_3L_{3-1}'L_{3-2}' \underset{k_{-19}}{\overset{k_{19}}{\rightleftarrows}} L_3 + L_{3-1}' + L_{3-2}'$

Derivatives:

$$\frac{dL_2}{dt} = k_{-8}[L_2T_2] - k_8[L_2][T_2] + k_{13}[L_2L_{2-1}'L_{2-2}'] - k_{-13}[L_2][L_{2-1}'][L_{2-2}']$$

$$\frac{dT_2}{dt} = k_{-8}[L_2T_2] - k_8[L_2][T_2] + k_9[L_2T_2][L_2'] - k_{-9}[L_2L_2'][T_2] + k_{-10}[T_2G_2G_3] - k_{10}[T_2][G_2][G_3]$$

$$\frac{dL_2T_2}{dt} = k_8[L_2][T_2] - k_{-8}[L_2T_2] + k_{-9}[L_2L_2'][T_2] - k_9[L_2T_2][L_2']$$

$$\frac{dL_2'}{dt} = k_{-9}[L_2L_2'][T_2] - k_9[L_2T_2][L_2']$$

$$\frac{dL_2L_2'}{dt} = k_9[L_2T_2][L_2'] - k_{-9}[L_2L_2'][T_2] + k_{-11}[L_2L_2'E] - k_{11}[L_2L_2'][E]$$

$$\frac{dG_2}{dt} = k_{-10}[T_2G_2G_3] - k_{10}[T_2][G_2][G_3]$$

$$\frac{dG_3}{dt} = k_{-10}[T_2G_2G_3] - k_{10}[T_2][G_2][G_3]$$

$$\frac{dT_2G_2G_3}{dt} = k_{10}[T_2][G_2][G_3] - k_{-10}[T_2G_2G_3]$$

$$\frac{dE}{dt} = k_{-11}[L_2L_2'E] - k_{11}[L_2L_2'][E] + k_{12}[L_2L_2'E] + k_{-17}[L_3L_3'E] - k_{17}[L_3L_3'][E] + k_{18}[L_3L_3'E]$$

$$\frac{dL_2L_2'E}{dt} = k_{11}[L_2L_2'][E] - k_{-11}[L_2L_2'E] - k_{12}[L_2L_2'E]$$

$$\frac{dL_2L_{2-1}'L_{2-2}'}{dt} = k_{12}[L_2L_2'E] - k_{13}[L_2L_{2-1}'L_{2-2}'] + k_{-13}[L_2][L_{2-1}'][L_{2-2}']$$

$$\frac{dL_{2-1}'}{dt} = k_{13}[L_2L_{2-1}'L_{2-2}'] - k_{-13}[L_2][L_{2-1}'][L_{2-2}']$$

$$\frac{dL_{2-2}'}{dt} = k_{13}[L_2L_{2-1}'L_{2-2}'] - k_{-13}[L_2][L_{2-1}'][L_{2-2}']$$

$$\frac{dL_3}{dt} = k_{-14}[L_3T_3] - k_{14}[L_3][T_3] + k_{19}[L_3L_{3-1}'L_{3-2}'] - k_{-19}[L_3][L_{3-1}'][L_{3-2}']$$

$$\frac{dT_3}{dt} = k_{-14}[L_3T_3] - k_{14}[L_3][T_3] + k_{15}[L_3T_3][L_3'] - k_{-15}[L_3L_3'][T_3] + k_{-21}[T_3M_1M_2][I_M] - k_{21}[T_3][M_1][M_2I_M]$$

$$\frac{dL_3T_3}{dt} = k_{14}[L_3][T_3] - k_{-14}[L_3T_3] + k_{-15}[L_3L_3'][T_3] - k_{15}[L_3T_3][L_3']$$

$$\frac{dM_2}{dt} = k_{-20}[M_2I_M] - k_{20}[M_2][I_M]$$

$$\frac{dI_M}{dt} = k_{-20}[M_2I_M] - k_{20}[M_2][I_M] + k_{21}[T_3][M_1][M_2I_M] - k_{-21}[T_3M_1M_2][I_M]$$

$$\frac{dM_2I_M}{dt} = k_{20}[M_2][I_M] - k_{-20}[M_2I_M] + k_{-21}[T_3M_1M_2][I_M] - k_{21}[T_3][M_1][M_2I_M]$$

$$\frac{dL_3'}{dt} = k_{-15}[L_3L_3'][T_3] - k_{15}[L_3T_3][L_3']$$

$$\frac{dL_3L_3'}{dt} = k_{15}[L_3T_3][L_3'] - k_{-15}[L_3L_3'][T_3] + k_{-17}[L_3L_3'E] - k_{17}[L_3L_3'][E]$$

$$\frac{dM_1}{dt} = k_{-21}[T_3M_1M_2][I_M] - k_{21}[T_3][M_1][M_2I_M]$$

$$\frac{dT_3M_1M_2}{dt} = k_{21}[T_3][M_1][M_2I_M] - k_{-21}[T_3M_1M_2][I_M]$$

$$\frac{dL_3L_3'E}{dt} = k_{17}[L_3L_3'][E] - k_{-17}[L_3L_3'E] - k_{18}[L_3L_3'E]$$

$$\frac{dL_3L_{3-1}'L_{3-2}'}{dt} = k_{18}[L_3L_3'E] - k_{19}[L_3L_{3-1}'L_{3-2}'] + k_{-19}[L_3][L_{3-1}'][L_{3-2}']$$

$$\frac{dL_{3-1}'}{dt} = k_{19}[L_3L_{3-1}'L_{3-2}'] - k_{-19}[L_3][L_{3-1}'][L_{3-2}']$$

$$\frac{dL_{3-2}'}{dt} = k_{19}[L_3L_{3-1}'L_{3-2}'] - k_{-19}[L_3][L_{3-1}'][L_{3-2}']$$

Figure S18. Computational simulation of the inhibitor I_M-gated transient DNAzymes (state R) shown in Figure 4. The kinetic scheme of the reactions associated with the time-dependent concentration changes during the dissipative transitions is summarized in the above equations. Knowing the time-dependent concentration changes of the complexes T_2/G_2+G_3 and T_3/M_1+M_2 during the dissipative transitions, we computationally simulated the time-dependent concentration changes by using Matlab R2019b.

Kinetic equations of the inhibitor I_G-gated transient DNAzymes (state S) shown in Figure 4:

(8) $L_2 + T_2 \underset{k_{-8}}{\overset{k_8}{\rightleftarrows}} L_2T_2$

(22) $G_2 + I_G \underset{k_{-22}}{\overset{k_{22}}{\rightleftarrows}} G_2I_G$

(9) $L_2T_2 + L_2' \underset{k_{-9}}{\overset{k_9}{\rightleftarrows}} L_2L_2' + T_2$

(23) $T_2 + G_2I_G + G_3 \underset{k_{-23}}{\overset{k_{23}}{\rightleftarrows}} T_2G_2G_3 + I_G$

(11) $L_2L_2' + E \underset{k_{-11}}{\overset{k_{11}}{\rightleftarrows}} L_2L_2'E$

(12) $L_2L_2'E \xrightarrow{k_{12}} E + L_2L_{2-1}'L_{2-2}'$

(13) $L_2L_{2-1}'L_{2-2}' \underset{k_{-13}}{\overset{k_{13}}{\rightleftarrows}} L_2 + L_{2-1}' + L_{2-2}'$

(14) $L_3 + T_3 \underset{k_{-14}}{\overset{k_{14}}{\rightleftarrows}} L_3T_3$

(15) $L_3T_3 + L_3' \underset{k_{-15}}{\overset{k_{15}}{\rightleftarrows}} L_3L_3' + T_3$

(16) $T_3 + M_1 + M_2 \underset{k_{-16}}{\overset{k_{16}}{\rightleftarrows}} T_3M_1M_2$

(17) $L_3L_3' + E \underset{k_{-17}}{\overset{k_{17}}{\rightleftarrows}} L_3L_3'E$

(18) $L_3L_3'E \xrightarrow{k_{18}} E + L_3L_{3-1}'L_{3-2}'$

(19) $L_3L_{3-1}'L_{3-2}' \underset{k_{-19}}{\overset{k_{19}}{\rightleftarrows}} L_3 + L_{3-1}' + L_{3-2}'$

Derivatives:

$$\frac{dL_2}{dt} = k_{-8}[L_2T_2] - k_8[L_2][T_2] + k_{13}[L_2L_{2-1}'L_{2-2}'] - k_{-13}[L_2][L_{2-1}'][L_{2-2}']$$

$$\frac{dT_2}{dt} = k_{-8}[L_2T_2] - k_8[L_2][T_2] + k_9[L_2T_2][L_2'] - k_{-9}[L_2L_2'][T_2] + k_{-23}[T_2G_2G_3][I_G] - k_{23}[T_2][G_2I_G][G_3]$$

$$\frac{dL_2T_2}{dt} = k_8[L_2][T_2] - k_{-8}[L_2T_2] + k_{-9}[L_2L_2'][T_2] - k_9[L_2T_2][L_2']$$

$$\frac{dG_2}{dt} = k_{-22}[G_2I_G] - k_{22}[G_2][I_G]$$

$$\frac{dI_G}{dt} = k_{-22}[G_2I_G] - k_{22}[G_2][I_G] + k_{23}[T_2][G_2I_G][G_3] - k_{-23}[T_2G_2G_3][I_G]$$

$$\frac{dG_2I_G}{dt} = k_{22}[G_2][I_G] - k_{-22}[G_2I_G] + k_{-23}[T_2G_2G_3][I_G] - k_{23}[T_2][G_2I_G][G_3]$$

$$\frac{dL_2'}{dt} = k_{-9}[L_2L_2'][T_2] - k_9[L_2T_2][L_2']$$

$$\frac{dL_2L_2'}{dt} = k_9[L_2T_2][L_2'] - k_{-9}[L_2L_2'][T_2] + k_{-11}[L_2L_2'E] - k_{11}[L_2L_2'][E]$$

$$\frac{dG_3}{dt} = k_{-23}[T_2G_2G_3][I_G] - k_{23}[T_2][G_2I_G][G_3]$$

$$\frac{dT_2G_2G_3}{dt} = k_{23}[T_2][G_2I_G][G_3] - k_{-23}[T_2G_2G_3][I_G]$$

$$\frac{dE}{dt}=k_{-11}[L_2L_2'E]-k_{11}[L_2L_2'][E]+k_{12}[L_2L_2'E]+k_{-17}[L_3L_3'E]-k_{17}[L_3L_3'][E]+k_{18}[L_3L_3'E]$$

$$\frac{dL_2L_2'E}{dt}=k_{11}[L_2L_2'][E]-k_{-11}[L_2L_2'E]-k_{12}[L_2L_2'E]$$

$$\frac{dL_2L_{2-1}'L_{2-2}'}{dt}=k_{12}[L_2L_2'E]-k_{13}[L_2L_{2-1}'L_{2-2}']+k_{-13}[L_2][L_{2-1}'][L_{2-2}']$$

$$\frac{dL_{2-1}'}{dt}=k_{13}[L_2L_{2-1}'L_{2-2}']-k_{-13}[L_2][L_{2-1}'][L_{2-2}']$$

$$\frac{dL_{2-2}'}{dt}=k_{13}[L_2L_{2-1}'L_{2-2}']-k_{-13}[L_2][L_{2-1}'][L_{2-2}']$$

$$\frac{dL_3}{dt}=k_{-14}[L_3T_3]-k_{14}[L_3][T_3]+k_{19}[L_3L_{3-1}'L_{3-2}']-k_{-19}[L_3][L_{3-1}'][L_{3-2}']$$

$$\frac{dT_3}{dt}=k_{-14}[L_3T_3]-k_{14}[L_3][T_3]+k_{15}[L_3T_3][L_3']-k_{-15}[L_3L_3'][T_3]+k_{-16}[T_3M_1M_2]-k_{16}[T_3][M_1][M_2]$$

$$\frac{dL_3T_3}{dt}=k_{14}[L_3][T_3]-k_{-14}[L_3T_3]+k_{-15}[L_3L_3'][T_3]-k_{15}[L_3T_3][L_3']$$

$$\frac{dL_3'}{dt}=k_{-15}[L_3L_3'][T_3]-k_{15}[L_3T_3][L_3']$$

$$\frac{dL_3L_3'}{dt}=k_{15}[L_3T_3][L_3']-k_{-15}[L_3L_3'][T_3]+k_{-17}[L_3L_3'E]-k_{17}[L_3L_3'][E]$$

$$\frac{dM_1}{dt}=k_{-16}[T_3M_1M_2]-k_{16}[T_3][M_1][M_2]$$

$$\frac{dM_2}{dt}=k_{-16}[T_3M_1M_2]-k_{16}[T_3][M_1][M_2]$$

$$\frac{dT_3M_1M_2}{dt}=k_{16}[T_3][M_1][M_2]-k_{-16}[T_3M_1M_2]$$

$$\frac{dL_3L_3'E}{dt}=k_{17}[L_3L_3'][E]-k_{-17}[L_3L_3'E]-k_{18}[L_3L_3'E]$$

$$\frac{dL_3L_{3-1}'L_{3-2}'}{dt}=k_{18}[L_3L_3'E]-k_{19}[L_3L_{3-1}'L_{3-2}']+k_{-19}[L_3][L_{3-1}'][L_{3-2}']$$

$$\frac{dL_{3-1}'}{dt}=k_{19}[L_3L_{3-1}'L_{3-2}']-k_{-19}[L_3][L_{3-1}'][L_{3-2}']$$

$$\frac{dL_{3-2}'}{dt}=k_{19}[L_3L_{3-1}'L_{3-2}']-k_{-19}[L_3][L_{3-1}'][L_{3-2}']$$

Figure S19. Computational simulation of the inhibitor I_G-gated transient DNAzymes (state S) shown in Figure 4. The kinetic scheme of the reactions associated with the time-dependent concentration changes during the dissipative transitions is summarized in the above equations. Knowing the time-dependent concentration changes of the complexes T_2/G_2+G_3 and T_3/M_1+M_2 during the dissipative transitions, we computationally simulated the time-dependent concentration changes by using Matlab R2019b.

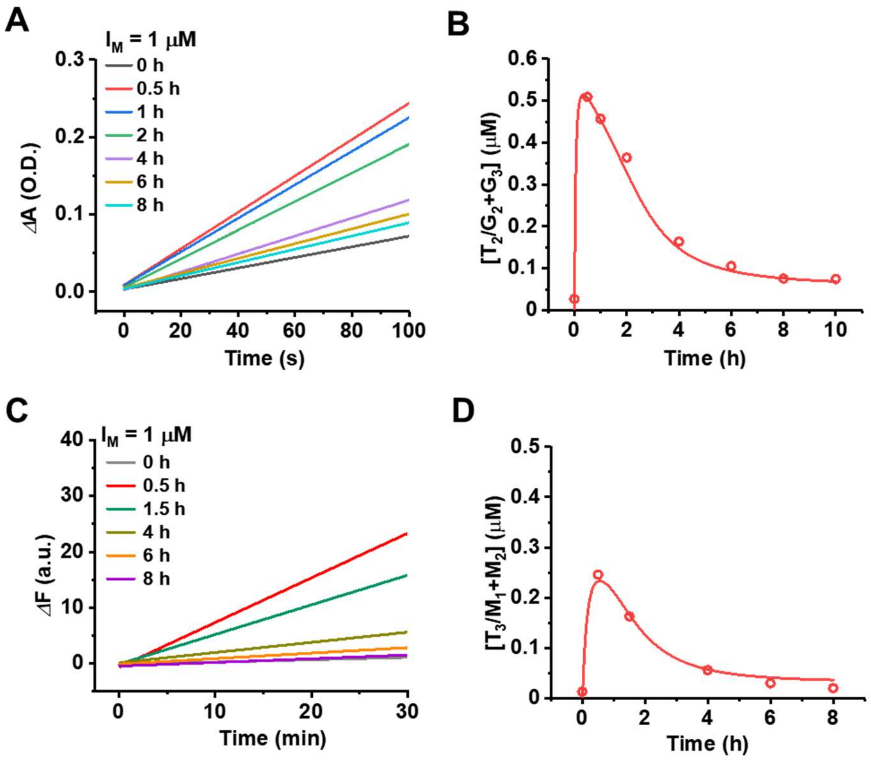

Figure S20. (A) Time-dependent absorbance changes of $ABTS^{\bullet-}$ formation catalyzed by the inhibitor I_M (1 μM)-gated transient DNAzymes (state R) at time intervals following addition of the triggers L_2' and L_3'. (B) Time-dependent concentration changes corresponding to the transient supramolecular T_2/G_2+G_3 hemin/G-quadruplex DNAzyme in the system. (C) Time-dependent fluorescence changes generated from the cleavage of S_1 by the inhibitor I_M (1 μM)-gated transient DNAzymes (state R) at time intervals following addition of triggers L_2' and L_3'. (D) Time-dependent concentration changes corresponding to the transient T_3/M_1+M_2 Mg^{2+}-ion-dependent DNAzyme in the system. In (B) and (D), dots correspond to the experimental data derived from the results shown in (A) and (C) by using the calibration curves in Figure S6 and S12, respectively. Solid curves correspond to the computationally simulated kinetic profiles using the kinetic models presented in Figure S18. In state R, L_2' = 4 μM, L_2' = 5 μM, Nt.BbvCI = 0.069 μM (T = 33 °C).

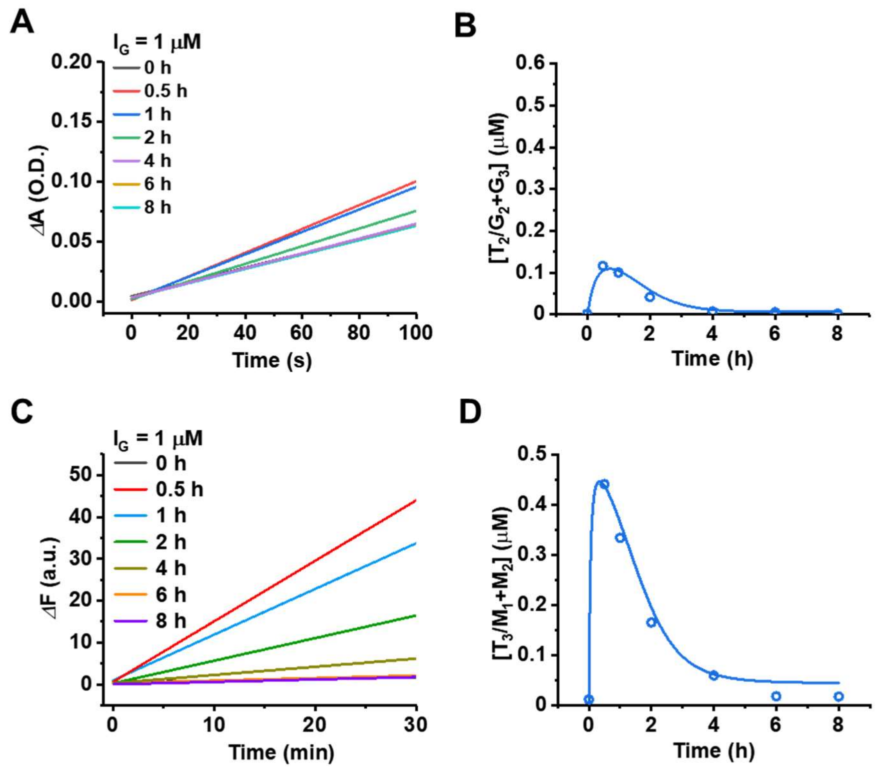

Figure S21. (A) Time-dependent absorbance changes of $ABTS^{\bullet-}$ formation catalyzed by the inhibitor I_G (1 μM)-gated transient DNAzymes (state S) at time intervals following addition of triggers L_2' and L_3'. (B) Time-dependent concentration changes corresponding to the transient supramolecular T_2/G_2+G_3 hemin/G-quadruplex DNAzyme in the system. (C) Time-dependent fluorescence changes generated from the cleavage of S_1 by the inhibitor I_G (1 μM)-gated transient DNAzymes (state S) at time intervals following addition of triggers L_2' and L_3'. (D) Time-dependent concentration changes corresponding to the transient T_3/M_1+M_2 Mg^{2+}-ion-dependent DNAzyme in the system. In (B) and (D), dots correspond to the experimental data derived from the results shown in (A) and (C) by using the calibration curves in Figure S6 and S12, respectively. Solid curves correspond to the computationally simulated kinetic profiles using the kinetic models presented in Figure S19. In state S, L_2' = 4 μM, L_2' = 5 μM, Nt.BbvCI = 0.069 μM (T = 33 °C).

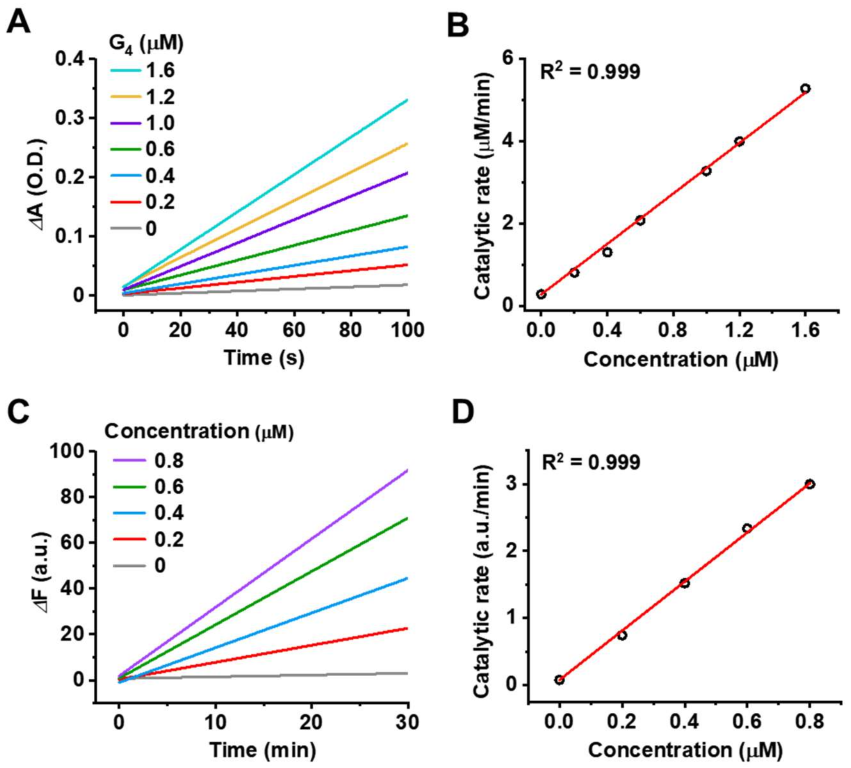

Figure S22. (A) The time-dependent absorbance changes of $ABTS^{\bullet-}$ formation catalyzed by variable concentrations of G_4-assembled hemin/G-quadruplex DNAzyme. (B) The derived calibration curve corresponding to the catalytic rate of $ABTS^{\bullet-}$ at different concentrations of G_4-assembled hemin/G-quadruplex DNAzyme. $ABTS^{2-}$ and H_2O_2 were 0.25 mM. (C) Time-dependent fluorescence changes generated from the cleavage of S_1 by the Mg^{2+}-ion-dependent DNAzyme upon the addition of variable concentrations of T_3 to bind M_3 and M_4. (D) The derived calibration curve corresponding to the catalytic rate at different concentrations of T_3/M_3+M_4 Mg^{2+}-ion-dependent DNAzyme.

Kinetic equations of the transient cascaded DNAzymes shown in Figure 6:

(24) $L_4 + T_4 \underset{k_{-24}}{\overset{k_{24}}{\rightleftarrows}} L_4T_4$

(25) $G_4 + C_4 \underset{k_{-25}}{\overset{k_{25}}{\rightleftarrows}} G_4C_4$

(26) $L_4T_4 + L_4' \underset{k_{-26}}{\overset{k_{26}}{\rightleftarrows}} L_4L_4' + T_4$

(27) $G_4C_4 + T_4 \underset{k_{-27}}{\overset{k_{27}}{\rightleftarrows}} T_4C_4 + G_4$

(28) $L_4L_4' + E \underset{k_{-28}}{\overset{k_{28}}{\rightleftarrows}} L_4L_4'E$

(29) $L_4L_4'E \xrightarrow{k_{29}} E + L_4L_{4-1}'L_{4-2}'$

(30) $L_4L_{4-1}'L_{4-2}' \underset{k_{-30}}{\overset{k_{30}}{\rightleftarrows}} L_4 + L_{4-1}' + L_{4-2}'$

(14) $L_3 + T_3 \underset{k_{-14}}{\overset{k_{14}}{\rightleftarrows}} L_3T_3$

(31) $L_3T_3 + G_4 \underset{k_{-31}}{\overset{k_{31}}{\rightleftarrows}} G_4L_3 + T_3$

(32) $T_3 + M_3 + M_4 \underset{k_{-32}}{\overset{k_{32}}{\rightleftarrows}} T_3M_3M_4$

(33) $G_4L_3 + E \underset{k_{-33}}{\overset{k_{33}}{\rightleftarrows}} G_4L_3E$

(34) $G_4L_3E \xrightarrow{k_{34}} E + L_3G_{4-1}G_{4-2}$

(35) $L_3G_{4-1}G_{4-2} \underset{k_{-35}}{\overset{k_{35}}{\rightleftarrows}} L_3 + G_{4-1} + G_{4-2}$

(36) $G_{4-2} + C_4 \underset{k_{-36}}{\overset{k_{36}}{\rightleftarrows}} G_{4-2}C_4$

Derivatives:

$$\frac{dL_4}{dt} = k_{-24}[L_4T_4] - k_{24}[L_4][T_4] + k_{30}[L_4L_{4-1}'L_{4-2}'] - k_{-30}[L_4][L_{4-1}'][L_{4-2}']$$

$$\frac{dT_4}{dt} = k_{-24}[L_4T_4] - k_{24}[L_4][T_4] + k_{26}[L_4T_4][L_4'] - k_{-26}[L_4L_4'][T_4] + k_{-27}[T_4C_4][G_4] - k_{27}[G_4C_4][T_4]$$

$$\frac{dL_4T_4}{dt} = k_{24}[L_4][T_4] - k_{-24}[L_4T_4] + k_{-26}[L_4L_4'][T_4] - k_{26}[L_4T_4][L_4']$$

$$\frac{dG_4}{dt} = k_{-25}[G_4C_4] - k_{25}[G_4][C_4] + k_{27}[G_4C_4][T_4] - k_{-27}[T_4C_4][G_4] - k_{31}[G_4][L_3T_3] + k_{-31}[T_3][G_4L_3]$$

$$\frac{dC_4}{dt} = k_{-25}[G_4C_4] - k_{25}[G_4][C_4] + k_{-36}[G_{4-2}C_4] - k_{36}[G_{4-2}][C_4]$$

$$\frac{dG_4C_4}{dt} = k_{25}[G_4][C_4] - k_{-25}[G_4C_4] + k_{-27}[T_4C_4][G_4] - k_{27}[G_4C_4][T_4]$$

$$\frac{dL_4'}{dt} = k_{-26}[L_4L_4'][T_4] - k_{26}[L_4T_4][L_4']$$

$$\frac{dL_4L_4'}{dt} = k_{26}[L_4T_4][L_4'] - k_{-26}[L_4L_4'][T_4] + k_{-28}[L_4L_4'E] - k_{28}[L_4L_4'][E]$$

$$\frac{dT_4C_4}{dt} = k_{27}[G_4C_4][T_4] - k_{-27}[T_4C_4][G_4]$$

$$\frac{dE}{dt}=k_{-28}[L_4L_4'E]-k_{28}[L_4L_4'][E]+k_{29}[L_4L_4'E]+k_{-33}[G_4L_3E]-k_{33}[G_4L_3][E]+k_{34}[G_4L_3E]$$

$$\frac{dL_4L_4'E}{dt}=k_{28}[L_4L_4'][E]-k_{-28}[L_4L_4'E]-k_{29}[L_4L_4'E]$$

$$\frac{dL_4L_{4-1}'L_{4-2}'}{dt}=k_{29}[L_4L_4'E]+k_{-30}[L_4][L_{4-1}'][L_{4-2}']-k_{30}[L_4L_{4-1}'L_{4-2}']$$

$$\frac{dL_{4-1}'}{dt}=k_{30}[L_4L_{4-1}'L_{4-2}']-k_{-30}[L_4][L_{4-1}'][L_{4-2}']$$

$$\frac{dL_{4-2}'}{dt}=k_{30}[L_4L_{4-1}'L_{4-2}']-k_{-30}[L_4][L_{4-1}'][L_{4-2}']$$

$$\frac{dL_3}{dt}=k_{-14}[L_3T_3]-k_{14}[L_3][T_3]+k_{35}[L_3G_{4-1}G_{4-2}]-k_{-35}[L_3][G_{4-1}][G_{4-2}]$$

$$\frac{dT_3}{dt}=k_{-14}[L_3T_3]-k_{14}[L_3][T_3]+k_{31}[G_4][L_3T_3]-k_{-31}[T_3][G_4L_3]+k_{-32}[T_3M_3M_4]-k_{32}[T_3][M_3][M_4]$$

$$\frac{dL_3T_3}{dt}=k_{14}[L_3][T_3]-k_{-14}[L_3T_3]+k_{-31}[T_3][G_4L_3]-k_{31}[G_4][L_3T_3]$$

$$\frac{dG_4L_3}{dt}=k_{31}[G_4][L_3T_3]-k_{-31}[T_3][G_4L_3]+k_{-33}[G_4L_3E]-k_{33}[G_4L_3][E]$$

$$\frac{dM_3}{dt}=k_{-32}[T_3M_3M_4]-k_{32}[T_3][M_3][M_4]$$

$$\frac{dM_4}{dt}=k_{-32}[T_3M_3M_4]-k_{32}[T_3][M_3][M_4]$$

$$\frac{dT_3M_3M_4}{dt}=k_{32}[T_3][M_3][M_4]-k_{-32}[T_3M_3M_4]$$

$$\frac{dG_4L_3E}{dt}=k_{33}[G_4L_3][E]-k_{-33}[G_4L_3E]-k_{34}[G_4L_3E]$$

$$\frac{dL_3G_{4-1}G_{4-2}}{dt}=k_{34}[G_4L_3E]-k_{35}[L_3G_{4-1}G_{4-2}]+k_{-35}[L_3][G_{4-1}][G_{4-2}]$$

$$\frac{dG_{4-1}}{dt}=k_{35}[L_3G_{4-1}G_{4-2}]-k_{-35}[L_3][G_{4-1}][G_{4-2}]$$

$$\frac{dG_{4-2}}{dt}=k_{35}[L_3G_{4-1}G_{4-2}]-k_{-35}[L_3][G_{4-1}][G_{4-2}]+k_{-36}[G_{4-2}C_4]-k_{36}[G_{4-2}][C_4]$$

$$\frac{dG_{4-2}C_4}{dt}=k_{36}[G_{4-2}][C_4]-k_{-36}[G_{4-2}C_4]$$

Figure S23. Computational simulation of the transient cascaded DNAzymes shown in Figure 6. The kinetic scheme of the reactions associated with the time-dependent concentration changes during the dissipative transitions is summarized in the above equations. Knowing the time-dependent concentration changes of G_4+G_{4-2} and T_3/M_3+M_4, during the dissipative transitions, we computationally simulated the time-dependent concentration changes by using Matlab R2019b.

Evaluation of the system displayed in Figure 2 in MDA-MB-231 cell lysate

The different systems described in the study operated, however, in homogeneous buffer solutions. As future applications of such networks are envisaged for therapeutic applications, the use of the artificial networks in native bioenvironments is an important goal. Towards the possible use of such artificial networks in native media, we examined the operation of the transient network displayed in **Figure 2** in a cancer cell lysate. The results and an accompanying discussion are presented in **Figure S24**.

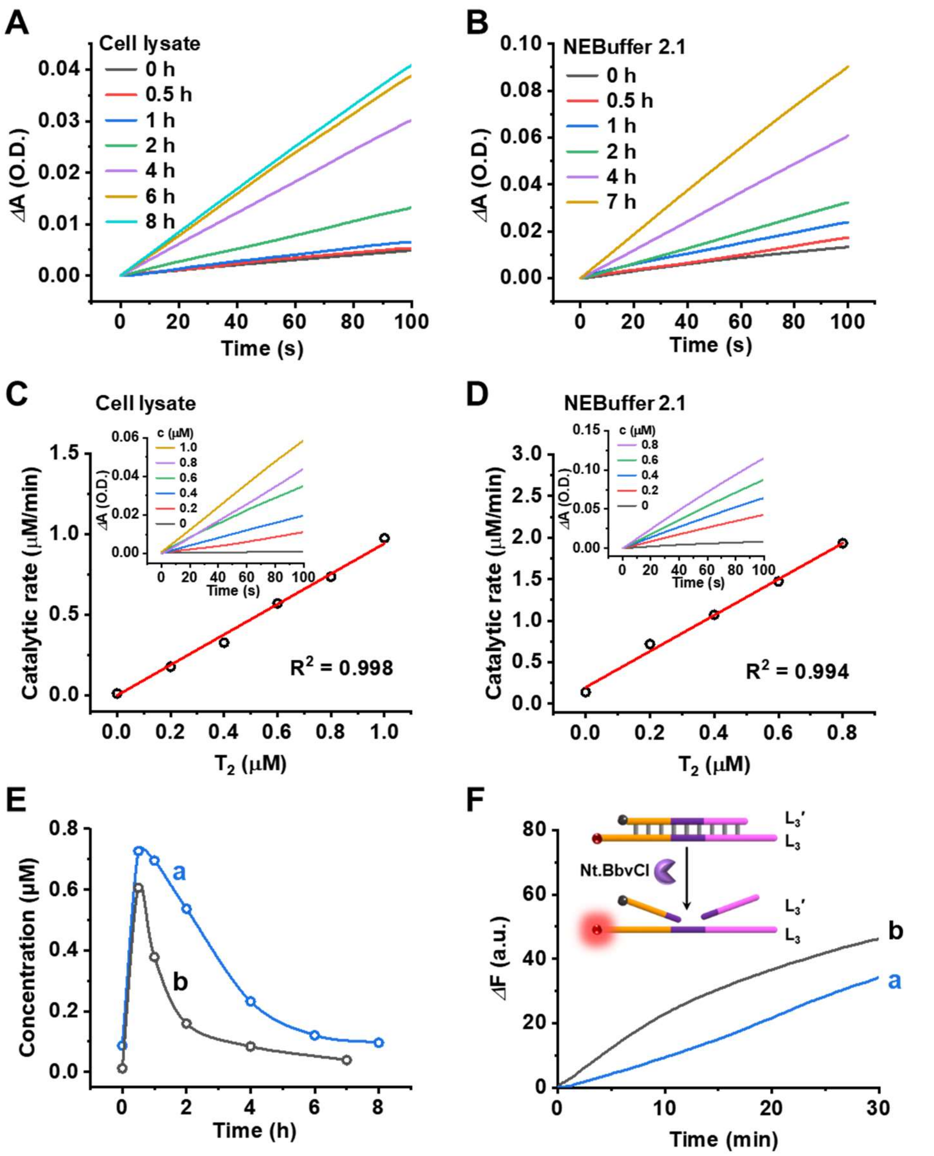

Figure S24. (A) Time-dependent absorbance changes of ABTS$^{\bullet-}$ catalyzed by the transient dissipative hemin/G-quadruplex DNAzyme at time intervals shown in Figure 2 in MDA-MB 231 breast cancer cell lysate. The cell lysate was prepared by lysing 10^6 MDA-MB 231 breast cancer cells in 1 mL of RIPA lysis buffer (50 mM Tris-HCl, 150 mM NaCl, 1% Triton X-100, 0.5% Sodium deoxylcholate, 0.1% SDS, 1 mM EDTA,

and 1 mM PMSF, pH 7.4 @25 °C). 10 mM $MgCl_2$ was added as a supplement. (B) Time-dependent absorbance changes of $ABTS^{\bullet-}$ catalyzed by the transient dissipative hemin/G-quadruplex DNAzyme at time intervals in 1 × NEBuffer 2.1 (50 mM sodium chloride, 10 mM tris-hydrochloride, 10 mM magnesium chloride, 100 μg/mL recombinant albumin, pH 7.9 @25 °C). (C) Calibration curve corresponding to the catalytic rate of $ABTS^{\bullet-}$ at different standard concentrations of T_2/G_2+G_3 hemin/G-quadruplex DNAzyme in cell lysate. Inset: The time-dependent absorbance changes of $ABTS^{\bullet-}$ upon the addition of variable concentrations of T_2 to bind G_2 and G_3. (C) Calibration curve corresponding to the catalytic rate of $ABTS^{\bullet-}$ at different standard concentrations of T_2/G_2+G_3 hemin/G-quadruplex DNAzyme in 1 × NEBuffer 2.1. Inset: The time-dependent absorbance changes of $ABTS^{\bullet-}$ upon the addition of variable concentrations of T_2 to bind G_2 and G_3. (E) The dotted curves show the transient concentrations of the hemin/G-quadruplex T_2/G_2+G_3 DNAzyme in (a) cell lysate, (b) 1 × NEBuffer 2.1. L_2/T_2 = 1 μM, G_2 = 1 μM, G_3 = 1 μM, hemin = 1 μM, Nt.BbvCI = 0.069 μM, L_1' = 4 μM. For all the UV measurements, the concentrations of $ABTS^{2-}$, H_2O_2, and KCl in final system was 0.25 mM, 0.50 mM and 25 mM KCl, respectively. (F) Schematic and fluorescence changes of Cy3-labeled L_3/BHQ2-labeled L_3' (0.5 μM) cleaved by Nt.BbvCI (0.023 μM) in (a) cell lysate, (b) 1 × NEBuffer 2.1.

Table S1. Rate constants derived from the computational simulation of the transient dissipative hemin/G-quadruplex DNAzyme shown in Figure 1 (T = 33 °C).

k_1	16.5 μM^{-1} min^{-1}	k_{-3}	7.0 μM^{-1} min^{-1}	k_6	2.0 min^{-1}
k_{-1}	1.0×10^{-4} min^{-1}	k_4	6.0×10^{-2} μM^{-1} min^{-1}	k_7	10.0 min^{-1}
k_2	14.0 μM^{-1} min^{-1}	k_{-4}	2.0×10^{-2} μM^{-1} min^{-1}	k_{-7}	2.0×10^{-2} μM^{-2} min^{-1}
k_{-2}	5.0×10^{-2} min^{-1}	k_5	25.0 μM^{-1} min^{-1}		
k_3	0.7 μM^{-1} min^{-1}	k_{-5}	5.0×10^{-3} min^{-1}		

Table S2. Rate constants derived from the computational simulation of the transient dissipative supramolecular hemin/G-quadruplex DNAzyme shown in Figure 2 (T = 33 °C).

k_8	20.0 μM^{-1} min^{-1}	k_{10}	8.0 μM^{-2} min^{-1}	k_{12}	2.0 min^{-1}
k_{-8}	2.0×10^{-4} min^{-1}	k_{-10}	3.0×10^{-2} min^{-1}	k_{13}	3.0 min^{-1}
k_9	5.0×10^{-2} μM^{-1} min^{-1}	k_{11}	25.0 μM^{-1} min^{-1}	k_{-13}	1.5×10^{-1} μM^{-2} min^{-1}
k_{-9}	9.0 μM^{-1} min^{-1}	k_{-11}	1.0×10^{-4} min^{-1}		

Table S3. Rate constants derived from the computational simulation of the transient dissipative Mg^{2+}-ion-dependent DNAzyme shown in Figure 3 (T = 33 °C).

k_{14}	16.0 μM^{-1} min^{-1}	k_{16}	2.2 μM^{-2} min^{-1}	k_{18}	2.0 min^{-1}
k_{-14}	5.0×10^{-4} min^{-1}	k_{-16}	3.0×10^{-2} min^{-1}	k_{19}	9.0 min^{-1}
k_{15}	5.0×10^{-2} μM^{-1} min^{-1}	k_{17}	25.0 μM^{-1} min^{-1}	k_{-19}	1.0×10^{-2} μM^{-2} min^{-1}
k_{-15}	10.0 μM^{-1} min^{-1}	k_{-17}	1.0×10^{-4} min^{-1}		

Experimentally obtained values: $k_{15} = 6.0\times10^{-2}$ μM^{-1} min^{-1}, $k_{-15} = 9.0$ μM^{-1} min^{-1}.

Table S4. Rate constants derived from the computational simulation of the parallel, non-gated transient DNAzymes (State Q) shown in Figure 4 (T = 33 °C).

k_8	20.0 μM^{-1} min^{-1}	k_{12}	2.0 min^{-1}	k_{-16}	3.0×10^{-2} min^{-1}
k_{-8}	2.0×10^{-4} min^{-1}	k_{13}	3.0 min^{-1}	k_{17}	25.0 μM^{-1} min^{-1}
k_9	5.0×10^{-2} μM^{-1} min^{-1}	k_{-13}	1.5×10^{-1} μM^{-2} min^{-1}	k_{-17}	1.0×10^{-4} min^{-1}
k_{-9}	9.0 μM^{-1} min^{-1}	k_{14}	16.0 μM^{-1} min^{-1}	k_{18}	2.0 min^{-1}
k_{10}	8.0 μM^{-2} min^{-1}	k_{-14}	5.0×10^{-4} min^{-1}	k_{19}	9.0 min^{-1}
k_{-10}	3.0×10^{-2} min^{-1}	k_{15}	5.0×10^{-2} μM^{-1} min^{-1}	k_{-19}	1.0×10^{-2} μM^{-2} min^{-1}
k_{11}	25.0 μM^{-1} min^{-1}	k_{-15}	10.0 μM^{-1} min^{-1}		
k_{-11}	1.0×10^{-4} min^{-1}	k_{16}	2.2 μM^{-2} min^{-1}		

Experimentally obtained values: $k_{15} = 6.0\times10^{-2}$ μM^{-1} min^{-1}, $k_{-15} = 9.0$ μM^{-1} min^{-1}.

Table S5. Rate constants derived from the computational simulation of the inhibitor I_M-gated transient DNAzymes (State R) shown in Figure 4 (T = 33 °C).

k_8	20.0 μM^{-1} min^{-1}	k_{12}	2.0 min^{-1}	k_{-15}	10.0 μM^{-1} min^{-1}
k_{-8}	2.0×10^{-4} min^{-1}	k_{13}	3.0 min^{-1}	k_{21}	0.2 μM^{-2} min^{-1}
k_9	5.0×10^{-2} μM^{-1} min^{-1}	k_{-13}	1.5×10^{-1} μM^{-2} min^{-1}	k_{-21}	0.1 μM^{-1} min^{-1}
k_{-9}	9.0 μM^{-1} min^{-1}	k_{14}	16.0 μM^{-1} min^{-1}	k_{17}	25.0 μM^{-1} min^{-1}
k_{10}	8.0 μM^{-2} min^{-1}	k_{-14}	5.0×10^{-4} min^{-1}	k_{-17}	1.0×10^{-4} min^{-1}
k_{-10}	3.0×10^{-2} min^{-1}	k_{20}	15.0 μM^{-1} min^{-1}	k_{18}	2.0 min^{-1}
k_{11}	25.0 μM^{-1} min^{-1}	k_{-20}	1.0×10^{-2} min^{-1}	k_{19}	9.0 min^{-1}
k_{-11}	1.0×10^{-4} min^{-1}	k_{15}	5.0×10^{-2} μM^{-1} min^{-1}	k_{-19}	1.0×10^{-2} μM^{-2} min^{-1}

Experimentally validated values: k_{15} = 6.0×10^{-2} μM^{-1} min^{-1}, k_{-15} = 9.0 μM^{-1} min^{-1}.

Table S6. Rate constants derived from the computational simulation of the inhibitor I_G-gated transient DNAzymes (State S) shown in Figure 4 (T = 33 °C).

k_8	20.0 μM^{-1} min^{-1}	k_{11}	25.0 μM^{-1} min^{-1}	k_{-15}	10.0 μM^{-1} min^{-1}
k_{-8}	2.0×10^{-4} min^{-1}	k_{-11}	1.0×10^{-4} min^{-1}	k_{16}	2.2 μM^{-2} min^{-1}
k_{22}	15.0 μM^{-1} min^{-1}	k_{12}	2.0 min^{-1}	k_{-16}	3.0×10^{-2} min^{-1}
k_{-22}	1.0 min^{-1}	k_{13}	3.0 min^{-1}	k_{17}	25.0 μM^{-1} min^{-1}
k_9	5.0×10^{-2} μM^{-1} min^{-1}	k_{-13}	1.5×10^{-1} μM^{-2} min^{-1}	k_{-17}	1.0×10^{-4} min^{-1}
k_{-9}	9.0 μM^{-1} min^{-1}	k_{14}	16.0 μM^{-1} min^{-1}	k_{18}	2.0 min^{-1}
k_{23}	8.0×10^{-2} μM^{-2} min^{-1}	k_{-14}	5.0×10^{-4} min^{-1}	k_{19}	9.0 min^{-1}
k_{-23}	0.1 μM^{-1} min^{-1}	k_{15}	5.0×10^{-2} μM^{-1} min^{-1}	k_{-19}	1.0×10^{-2} μM^{-2} min^{-1}

Experimentally validated values: k_{15} = 6.0×10^{-2} μM^{-1} min^{-1}, k_{-15} = 9.0 μM^{-1} min^{-1}.

Table S7. Rate constants derived from the computational simulation of the transient cascaded DNAzymes shown in Figure 6 (T = 33 °C).

k_{24}	10.0 μM^{-1} min^{-1}	k_{-28}	1.0×10^{-4} min^{-1}	k_{-32}	0.5 min^{-1}
k_{-24}	1.0×10^{-6} min^{-1}	k_{29}	2.0 min^{-1}	k_{33}	25.0 μM^{-1} min^{-1}
k_{25}	12.0 μM^{-1} min^{-1}	k_{30}	1.8 min^{-1}	k_{-33}	1.0×10^{-4} min^{-1}
k_{-25}	1.5×10^{-2} min^{-1}	k_{-30}	5.0×10^{-2} μM^{-2} min^{-1}	k_{34}	2.0 min^{-1}
k_{26}	8.0×10^{-3} μM^{-1} min^{-1}	k_{14}	16.0 μM^{-1} min^{-1}	k_{35}	0.05 min^{-1}
k_{-26}	7.0 μM^{-1} min^{-1}	k_{-14}	5.0×10^{-4} min^{-1}	k_{-35}	5.0×10^{-4} μM^{-2} min^{-1}
k_{27}	4.5 μM^{-1} min^{-1}	k_{31}	1.8×10^{-2} μM^{-1} min^{-1}	k_{36}	12.0 μM^{-1} min^{-1}
k_{-27}	9.0×10^{-2} μM^{-1} min^{-1}	k_{-31}	1.0 μM^{-1} min^{-1}	k_{-36}	3.0×10^{-3} min^{-1}
k_{28}	25.0 μM^{-1} min^{-1}	k_{32}	1.8 μM^{-2} min^{-1}		
